# Extending the utility of [Pd(NHC)(cinnamyl)Cl] precatalysts: Direct arylation of heterocycles

**DOI:** 10.3762/bjoc.8.187

**Published:** 2012-09-27

**Authors:** Anthony R Martin, Anthony Chartoire, Alexandra M Z Slawin, Steven P Nolan

**Affiliations:** 1 EaStCHEM School of Chemistry, University of St Andrews, North Haugh, St Andrews, KY16 9ST, UK

**Keywords:** C–H functionalization, direct arylation, heterocycles, N-heterocyclic carbenes, palladium

## Abstract

The use of [Pd(NHC)(cinnamyl)Cl] precatalysts in the direct arylation of heterocycles has been investigated. Among four different precatalysts, [Pd(SIPr)(cinnamyl)Cl] proved to be the most efficient promoter of the reaction. The C–H functionalization of sulfur- or nitrogen-containing heterocycles has been achieved at low catalyst loadings. These catalyst charges range from 0.1 to 0.01 mol % palladium.

## Introduction

As a powerful addition to the classic palladium cross-coupling reactions, C–H bond functionalization has become a growing field of research over the last few years. The ubiquity of C–H bonds makes them a convenient and cost-effective anchoring position within viable substrates, as no derivatisation to form an organometallic reagent is required. Moreover, among the plethora of C–H bonds present on a molecule, it is often possible to target one C–H linkage specifically, taking advantage of directing groups or particular catalyst selectivity [1–5]. Thus, heteroaromatic scaffolds, which are a common feature in biologically relevant compounds and in materials science [6–7] can be selectively arylated as the heteroatom can act as an intrinsic orientating group [8].

Despite the efficiency of well-defined palladium catalysts bearing NHC (N-heterocyclic carbene) ancillary ligands in classical cross-coupling reactions, they have rarely been applied to direct arylation procedures [9–16]. Among the family of [Pd(NHC)] complexes, the [Pd(NHC)(cin)Cl] (cin = cinnamyl) species are known for their ease of activation through the reduction of the metal centre from Pd(II) to Pd(0) [17]. Therefore, we have investigated the use of such precatalysts in the direct arylation of heteroaromatic compounds in order to compare them to ligand-free or phosphine-bearing catalytic systems, and in the end to see whether the reactivity and application scope of these commercially available complexes could be broadened to include C–H bond functionalization transformations.

**Figure 1:**
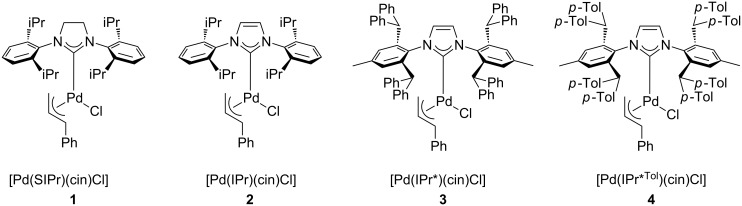
[Pd(NHC)(cin)Cl] catalysts examined in direct arylation.

We now report the activity of the [Pd(NHC)(cin)Cl] complexes **1–4** in the direct arylation of heterocycles with NHC ligands being SIPr (1,3-bis(2,6-diisopropylphenyl)-4,5-dihydroimidazol-2-ylidene), IPr (1,3-bis(2,6-diisopropylphenyl)imidazol-2-ylidene), IPr* (1,3-bis(2,6-bis(diphenylmethyl)-4-methylphenyl)imidazol-2-ylidene) and IPr*^Tol^ (1,3-bis(2,6-bis(di-*p*-tolylmethyl)-4-methylphenyl)imidazol-2-ylidene) (Figure 1). Complexes **1** and **2** are commercially available and have proven to be highly efficient in Suzuki–Miyaura coupling and Buchwald–Hartwig amination reactions [17–20]. We have also evaluated the recently reported [Pd(IPr*)(cin)Cl] (**3**), which has shown potency in Suzuki–Miyaura couplings [21] and Buchwald–Hartwig N-arylations [22] even with challenging substrates. To complete this study and to examine the effect of bulky ligands about the metal centre, we have synthesised a new complex [Pd(IPr*^Tol^)(cin)Cl] (**4**), which is a IPr* congener.

## Results and Discussion

**Scheme 1:**
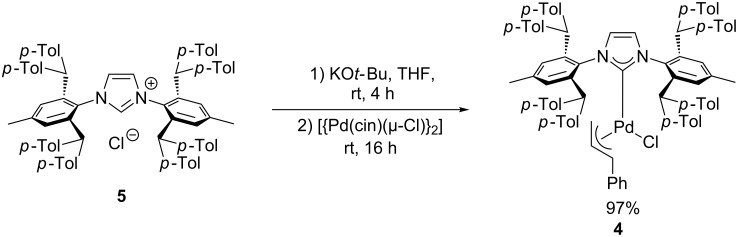
Synthesis of [Pd(IPr*^Tol^)(cin)Cl] (**4**).

The study begins with the preparation of the palladium complex **4**. Following the strategy recently reported by Markó [23], we were successful in the synthesis of the IPr*^Tol^·HCl imidazolium salt **5** in a 53% overall yield (see Supporting Information File 1). Subsequently, **5** was treated with KO*t*-Bu in dry THF to generate the corresponding free carbene in situ. The expected [Pd(IPr*^Tol^)(cin)Cl] was then obtained in an excellent yield (97%) by a simple fragmentation of the palladium dimer [{Pd(cin)(µ-Cl)}_2_] using the free carbene solution (Scheme 1).

**Figure 2:**
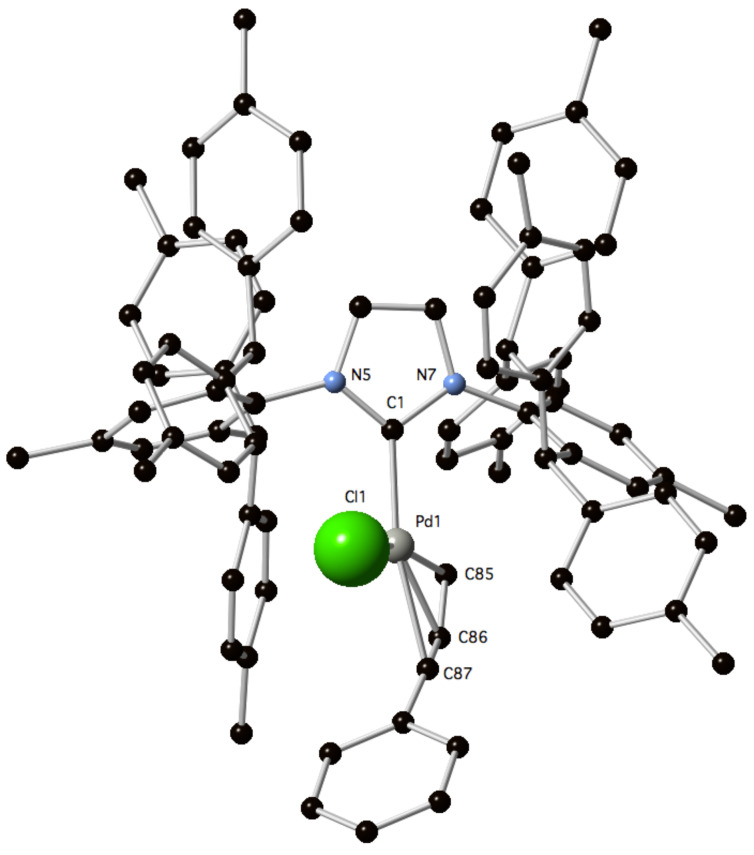
Molecular structure of **4**. H atoms were omitted for clarity. Selected bond lengths (Å) and angles (°): Pd1–C1 2.034(0), Pd1–Cl1 2.352(5), Pd1–C85 2.132(8), Pd1–C86 2.119(7), Pd1–C87 2.226(6); C1–Pd1-C85 102.9(5), C85–Pd1–C87 71.2(6), C87–Pd1–Cl1 93.3(8), Cl1–Pd1–C1 91.8(6).

The newly synthesized complex **4** was unequivocally characterised by X-ray diffraction [24] (Figure 2, Supporting Information File 2 and Supporting Information File 3) after suitable crystals were grown from slow diffusion of hexane in dichloromethane. Based on this crystal structure, the percentage buried volume (%*V*_Bur_) of the IPr*^Tol^ ancillary ligand was determined by using the “Samb*V*ca” web application [25] and compared to complexes **1**–**3** (Table 1) [21]. IPr*^Tol^ featured a %*V*_Bur_ in the same range as IPr* (+0.4% difference). SIPr and IPr have been reported as less hindered ligands with %*V*_Bur_ of 37.0 and 36.7, respectively. The length of the Pd–C1 bond in **4** was also examined and is close to the one observed in **3**.

**Table 1:** Comparison of the %*V*_Bur_ and d(Pd–C1) in the [Pd(NHC)(cin)Cl] family.

NHC	%*V_B_*_ur_^a^	Pd–C1 (Å)

SIPr	37.0	2.025(7)
IPr	36.7	2.041(9)
IPr*	44.6	2.038(6)
IPr*^Tol^	45.0	2.034(0)

^a^%*V*_Bur_ calculated for a 2.00 Å Pd–C1 length.

**Figure 3:**
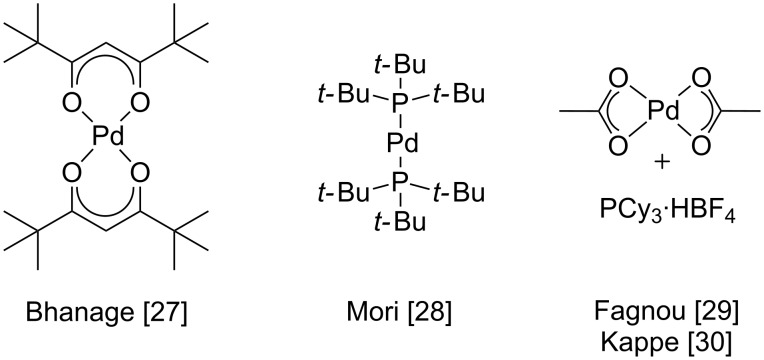
Previously reported catalytic systems in the direct arylation of benzothiophene (**6**).

With complexes **1**–**4** in hand, their catalytic activity towards the direct arylation of heteroaromatic compounds was evaluated. For this purpose, the arylation of benzothiophene (**6**) with 4-bromotoluene (**7**) was selected as a benchmark reaction (Table 2). This C–H functionalization, initially described by Ohta [26], was then reported by Bhanage and Mori using 2–10 mol % of well-defined palladium catalysts [27–28] (Figure 3). Alternatively, Fagnou and Kappe proposed a Pd/phosphine system involving 1–2 mol % of palladium and 2–4 mol % of phosphine [29–30], but no example of this reaction involving a well-defined [Pd(NHC)] complex has been described. However, it is noteworthy that variously substituted benzothiophene cores have been extensively studied in the direct arylation process [4,31–37].

**Table 2:** Catalyst screening for the direct arylation of benzothiophene (**6**).

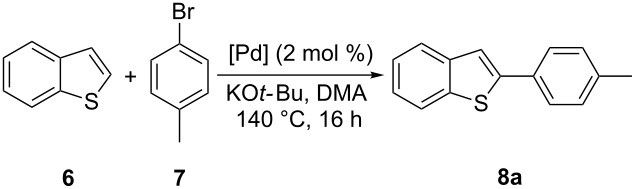	

Catalyst	Conversion (%)^a^

[Pd(SIPr)(cin)Cl] (**1**)	76
[Pd(IPr)(cin)Cl] (**2**)	50
[Pd(IPr*)(cin)Cl] (**3**)	8
[Pd(IPr*^Tol^)(cin)Cl] (**4**)	49

^a^Conversion of the starting material into C–H arylated product determined by GC, [**6**] = 0.3 M.

Initial screening of precatalysts **1**–**4** was performed with a 2 mol % loading, by using KO*t*-Bu as the base, which is known to efficiently activate the [Pd(NHC)(cin)Cl] precatalysts [17]. DMA was selected as the solvent and the reaction was conducted at 140 °C.

This survey showed that **1** is the most efficient precatalyst under these reaction conditions with 76% conversion of the starting material. Precatalysts **2** and **4** exhibited closely related activity, with 50 and 49% conversion, respectively. However, complex **3** gave relatively poor conversion of the benzothiophene (**6**).

Thus, selecting **1** as the best precatalyst, the use of other solvents, bases and additives was evaluated to optimize the reaction (see the Supporting Information File 1). From this optimization study, it was found that 0.1 mol % of **1** with K_2_CO_3_ in DMA as solvent at 140 °C in the presence of a catalytic amount of pivalic acid (30 mol %) generated the best reaction conditions. Under these optimized parameters, a second precatalyst screening was performed. As shown in Table 3, better activity was observed for precatalysts **1** and **2**, which have smaller ligands when compared to the NHCs in **3** and **4**. This result suggests a strong dependence of the activity on the steric properties of the NHC ligand. Moreover, the small difference between **1** and **2** underlines the fact that the difference in the σ-donation properties of the NHC ligands [38–41] is not likely to play a crucial role in the catalytic activity.

**Table 3:** Catalyst screening under optimised conditions.

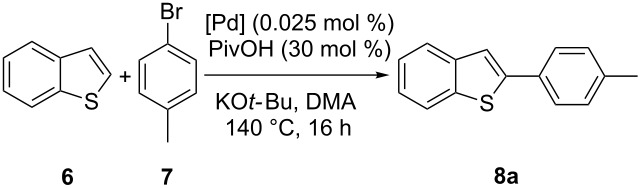	

Catalyst	Conversion (%)^a^

[Pd(SIPr)(cin)Cl] (**1**)	80
[Pd(IPr)(cin)Cl] (**2**)	75
[Pd(IPr*)(cin)Cl] (**3**)	57
[Pd(IPr*^Tol^)(cin)Cl] (**4**)	58

^a^Conversion of the starting material into C–H arylated product determined by GC, [**6**] = 0.3 M.

In comparison with the previously mentioned methodologies to perform this C–H functionalization [27–30], the catalyst loading can be decreased by at least 10-fold without drastically affecting the yield (Table 4, entry 1). Using the optimized reaction conditions, we examined the scope and the limitations of this catalytic system using various aryl bromides and heterocycles (Table 4). It appeared that the sterics of the aryl bromide had almost no impact on the reaction. Indeed, *para-*, *meta-* and *ortho-* substituted aryl bromides could be employed to arylate **6** in good yields. (Table 4, entries 1–3, 77–89%). However, *ortho*-disubstituted aryl bromide, such as bromomesitylene appeared to be too sterically demanding and led to no conversion (data not shown). Concerning the electronic properties of the aryl bromide, electron-withdrawing (EWG) and electron-donating groups (EDG) were tolerated, although the presence of EWGs resulted in decreased yields (Table 4, entries 4–6, 49–70%). The substrate 4-bromobenzaldehyde was also successfully involved in the direct arylation of **6**. Despite its electron-withdrawing nature as well as its high reactivity, the expected biaryl was obtained in moderate yield (Table 4, entry 7). The limits of the scope were determined by switching from benzothiophene (**6**) to the more sterically demanding 3-methylbenzothiophene (**9**) (Table 4, entries 8–10). Closely related reactivity was observed for **6** and **9**, as these were arylated in comparable yields (Table 4, entry 1 vs 8, 3 vs 9 and 6 vs 10).

**Table 4:** Palladium-NHC catalysed direct arylation of heterocycles with arylbromides.

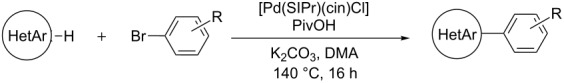				

Entry^a^	Heterocycles	Products	R	Yield^b^

1^c^	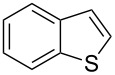 **6**	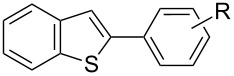 **8**	4-Me, **8a**	89%
2^c^			3-Me, **8b**	80%
3^c^			2-Me, **8c**	77%
4^c^			4-OMe, **8d**	70%
5^c^			4-Cl, **8e**	49%
6^c^			4-F, **8f**	53%
7^c^			4-CHO, **8g**	37%

8	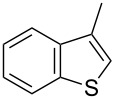 **9**	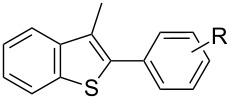 **10**	4-Me, **10a**	85%
9			2-Me, **10b**	83%
10			4-F, **10c**	52%

11	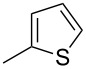 **11**	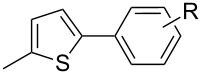 **12**	4-Me, **12a**	90%
12			4-OMe, **12b**	75%
13			4-F, **12c**	57%

14	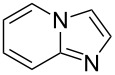 **13**	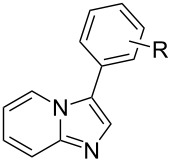 **14**	4-Me, **14a**	74%
15^d^				69%
16^d^			4-OMe, **14b**	53%
17^d^			4-F, **14c**	76%

^a^Unless noted, reactions were performed on 0.6 mmol scale with: Heterocycle (1 equiv), aryl bromide (1 equiv), [Pd(SIPr)(cin)Cl] (0.1 mol %), PivOH (30 mol %), K_2_CO_3_ (1.5 equiv), DMA (2 mL), 140 °C. ^b^Isolated yields, average of two independent runs. ^c^**6** (1.2 equiv). ^d^[Pd(SIPr)(cin)Cl] (0.01 mol %).

A more challenging heterocycle, 2-methylthiophene (**11**), was investigated. Simple thiophene rings are known to be less reactive in C–H functionalization reactions [42]; nevertheless, **11** was successfully arylated in moderate to good yields, depending on the electronic properties of the bromobenzene substituents (Table 4, entries 11–13, 57–90%). Electron rich 4-methoxybromobenzene reacted more efficiently than the electron poor 4-fluorobromobenzene. An opposite effect of the electronics was observed by Doucet et al. in their ligandless procedure at low catalyst loadings [43–44]. This is surely due to the nature of the catalyst and thus offers complementary direct arylation methods for thiophene derivatives.

To complete the study, experiments were performed at lower catalyst loading using imidazopyridine (**13**). This class of substrate has recently been involved, by Doucet et al. [45], in direct arylation with a catalytic charge of Pd(OAc)_2_ ranging from 0.1 to 0.01 mol %. In our case, comparable yields were obtained when the catalyst loading was decreased from 0.1 to 0.01 mol %, highlighting the high efficiency of the catalytic system (Table 4, entries 14 and 15). Following the same trend as reported by Doucet [45], a better reactivity was observed with bromobenzenes substituted with EWGs compared to with EDGs (Table 4, entries 16 and 17).

## Conclusion

In summary, we report here the synthesis and characterization of a new member of the [Pd(NHC)(cin)Cl] family, [Pd(IPr*^Tol^)(cin)Cl]. The catalytic activity of this family of complexes was surveyed in the direct arylation of heterocycles. The bulkiness of the NHC ligand appears to play a major role in the catalytic efficiency, whereas the σ-donation properties (within the small electronic space examined) have little influence. Among the four complexes, [Pd(SIPr)(cin)Cl] exhibited the highest catalytic efficiency and was investigated for the arylation of various benzothiophenes, thiophene and imidazopyridine. C–H functionalization of such heterocycles was performed in moderate to good yields by using only 0.1–0.01 mol % of precatalyst. This study highlights the fact that [Pd(NHC)(cin)Cl] complexes are multipurpose precatalysts as they may be utilised in various cross-coupling and, now, C–H-bond-functionalization reactions.

## Experimental

### General procedure for the direct arylation of heterocycles

In a glovebox, a vial containing a stirring bar was charged with K_2_CO_3_ (124 mg, 0.9 mmol, 1.5 equiv) and pivalic acid (0.18 mmol, 18 mg, 30 mol %), and sealed with a screw cap fitted with a septum. The heterocycle (0.6 mmol, 1.0 equiv) and/or the arylbromide (0.6 mmol, 1.0 equiv) were added at this point if in solid form, and DMA (1.9 mL) was poured into the vial. Outside of the glovebox, the heterocycle and/or the aryl bromide were added at this point if in liquid form. Finally, [Pd(SIPr)(cin)Cl] (**1**) was added as a 0.06 M solution in DMA (0.6–6 µmol, 10–100 µL, 0.01–0.1 mol %), and the vial was heated to 140 °C for 16 h. The solution was then cooled down to room temperature, diluted with 40 mL of ethyl acetate, and washed with water (2 × 20 mL) and brine (20 mL). The organic layer was dried over MgSO_4_, filtered and concentrated in vacuo. The crude residue was finally purified by either trituration in pentane (if not soluble) or silica-gel column chromatography using pentane as the eluent.

## Supporting Information

File 1 Synthesis and characterization of complex **4**; compound characterization data for all the direct arylated products and copies of their ^1^H and ^13^C NMR spectra.

File 2 CIF-Check for compound **4**.

File 3 Crystal structure data for compound **4**.
