# Supplementary material for: Extending the utility of [Pd(NHC)(cinnamyl)Cl] precatalysts: Direct arylation of heterocycles
Source: Beilstein J Org Chem. 2012 Sep 27;8:1637–43. doi: 10.3762/bjoc.8.187 (PMC3510996; doi:10.3762/bjoc.8.187)
Supplement: File 1 — Synthesis and characterization of complex 4; compound characterization data for all the direct arylated products and copies of their 1H and 13C NMR spectra. [file Beilstein_J_Org_Chem-08-1637-s001.pdf]

Supporting Information

for

Extending the utility of [Pd(NHC)(cinnamyl)Cl] precatalysts: Direct arylation of heterocycles

Anthony R. Martin, Anthony Chartoire, Alexandra M. Z. Slawin and Steven P. Nolan*

Address: EastCHEM School of Chemistry, University of St Andrews, North Haugh, St Andrews, KY16 9ST, UK

Email: Steven P. Nolan* - snolan@st-andrews.ac.uk

* Corresponding author

Synthesis and characterization of complex 4; compound characterization data for all the direct arylated products and copies of their ^{1}H and ^{13}C NMR spectra

Table of contents

General considerations S2

Synthesis and characterisation of 4 S3–6

Optimisation reactions S7–8

General procedure for the direct arylation of heterocycles S9

Characterisation data of compounds 8a–g, 10a–c, 12a–c and 14a–c S9–16

References S16

^{1}H and ^{13}C NMR spectra of all compounds S17–36

General considerations:

All aryl halides and heterocycles were used as received. Anhydrous solvents (1,4-dioxane, tetrahydrofuran (THF), dimethylacetamide (DMA), dimethylformamide (DMF) and toluene) and the bases (NaO*t*-Bu, KO*t*-Bu, Na_2CO_3, K_2CO_3, Cs_2CO_3, KOH, LiHMDS) were stored in a glovebox and used as received. [{Pd(cinnamyl)(μ-Cl)}$_2$] is commercially available (UMICORE) and was stored in a glovebox.

Flash chromatography was performed on silica gel 60 Å pore diameter and 40–63 μm particle size.

^{1}H and ^{13}C Nuclear Magnetic Resonance (NMR) spectra were recorded on a Bruker-300 MHz or 400 MHz spectrometer at ambient temperature in CD_2Cl_2 or $CDCl_3$. Chemical shifts (δ) are reported in ppm, relative to the solvent residual peak CD_2Cl_2 (5.32 ppm and 54.00 ppm) and $CDCl_3$ (7.26 ppm and 77.16 ppm). Data for ^{1}H NMR are reported as follows: chemical shift, multiplicity (s = singlet, d = doublet, t = triplet, br = broad signal, m = multiplet), coupling constants (*J*) in hertz and integration.

Elemental analyses were performed at London Metropolitan University 166–220 Holloway Road, London, N7 8DB.

Gas chromatography analyses (GC) were performed on an Agilent 7890A apparatus equipped with a flame ionization detector and a (5%-phenyl)methylpolysiloxane column (30 m, 320 μm, film: 0.25 μm). Flow rate 1 mL/min constant flow, inlet temperature 260 °C, column temperature 50 °C, 20 °C/min increase to 300 °C (held for 1 min), total time 7.6 min.

Mass spectrometry was performed by the EPSRC National Mass Spectrometry Service Centre at Swansea University, Grove building, Singleton Park, Swansea, SA2 8PP, Wales, UK

Synthesis of [Pd(IPr*^{Tol})(cin)Cl] 4:

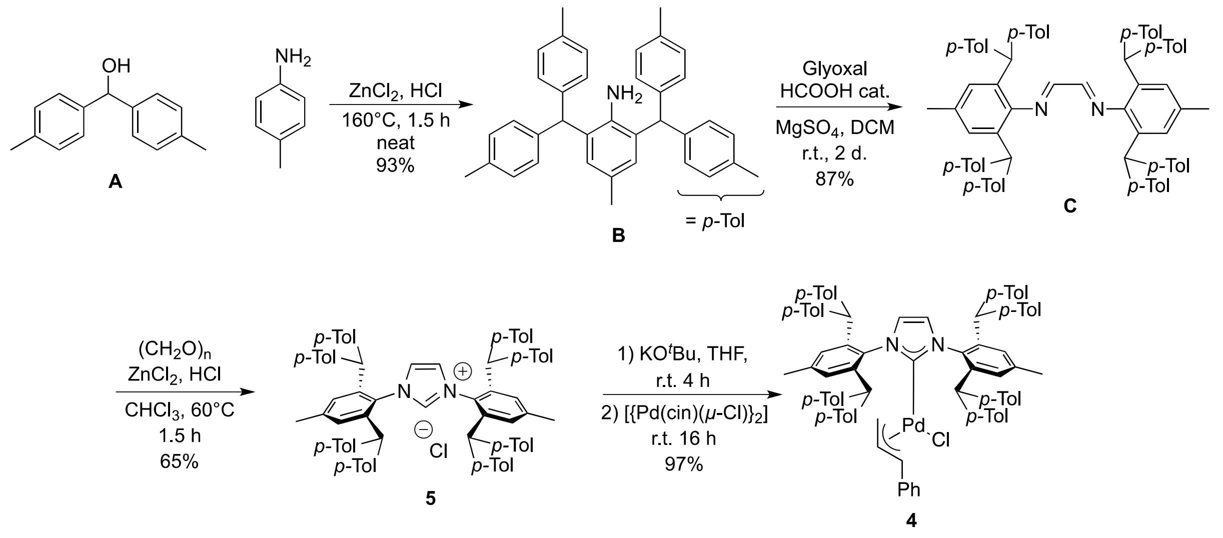

<u>Synthesis of the 2,6-bis(di-*p*-tolylmethyl)-4-methylaniline (**B**)</u>:

In a 250 mL round-bottomed flask, *p*-toluidine (3.34 g, 32 mmol) and di-*p*-tolylmethanol (**A**) [1] (13.58g, 64 mmol) were stirred together at 160 °C until liquid. A solution of zinc chloride (2.18 g, 16 mmol) in HCl 36% (2.7 mL) was added dropwide to the reaction mixture. The flask was then opened to allow water evaporation, and stirring was continued at 160 °C until solidification of the reacting media (1.5 h). The residue was then allowed to cool to room temperature, dissolved in CH_2Cl_2 (300 mL), and washed with NH_4Cl aq. saturated solution and brine. The organic layer was subsequently dried over K_2CO_3, and 20 g of silica gel was added to it. After filtration and concentration in *vacuo*, the 2,6-bis(di-*p*-tolylmethyl)-4-methylaniline (**B**) was obtained as an off-white powder (14.77 g, 93%), which did not need further purification.

^{1}H NMR ($CDCl_3$, 400 MHz) δ 2.09 (s, 3H, $CH_{3\text{-Tol}}$), 2.37 (s, 12H, $CH_{3\text{-Tol}}$), 3.37 (br, 2H, NH_2), 5.43 (s, 2H, C*H*), 6.46 (s, 2H, H_{Ar}), 7.04 (d, *J* 8.2 Hz, 8H, H_{Ar}), 7.13 (d, *J* 7.9 Hz, 8H, H_{Ar}).

^{13}C NMR ($CDCl_3$, 75 MHz) δ 21.2 (*C*H_3), 21.3 (*C*H_3), 51.8 (*C*H), 126.6 (C_{Ar}), 129.0 (CH_{Ar}), 129.3 (CH_{Ar}), 129.5 (CH_{Ar}), 129.6 (C_{Ar}), 136.1 (C_{Ar}), 139.8 (C_{Ar}) 140.2 (C_{Ar}).

HRMS $(NESI)^+$: Calcd for $C_{37}H_{38}N$: 495.2999, found: 495.2994.

Synthesis of the *N,N'*-bis(2,6-bis(di-*p*-tolylmethyl)-4-methylphenyl)diazabutadiene (**C**):

To a solution of aniline **B** (14.45 g, 29.15 mmol) dissolved in 300 mL of CH_2Cl_2, was added $MgSO_4$ (15 g), glyoxal (40 wt % in H_2O, 3.32 mL, 29.12 mmol) and a catalytic amount of formic acid (5 drops). The reacting mixture was stirred at room temperature for 2 days prior to filtration. The cake was washed profusely with CH_2Cl_2 until the filtrate became colourless. The latter was then concentrated to dryness and the residue was suspended in ethyl acetate. The slurry was finally filtered and washed with ethyl acetate (2 × 50 mL) to afford **C** as a bright yellow powder (12.88 g, 87%), which was dried under high vacuum.

^1H NMR (CD_2Cl_2, 400 MHz) δ 2.12 (s, 6H, $CH_{3\text{-Tol}}$), 2.27 (s, 24H, $CH_{3\text{-Tol}}$), 5.15 (s, 4H, C*H*), 6.68 (s, 4H, H_{Ar}), 6.85 (d, *J* 7.9 Hz, 16H, H_{Ar}), 7.02 (d, *J* 8.1 Hz, 16H, H_{Ar}), 7.28 (s, 2H, H_{imine}).

^{13}C NMR (CD_2Cl_2, 75 MHz) δ 21.3 (*C*H_3), 21.6 (*C*H_3), 50.9 (*C*H), 129.3 (C_{Ar}), 129.5 (CH_{Ar}), 129.8 (CH_{Ar}), 132.8 (C_{Ar}), 134.0 (C_{Ar}), 136.4 (CH_{Ar}), 141.5 (CH_{Ar}) 147.3 (C_{Ar}), 164.4 (*C*=N).

HRMS $(NESI)^+$: Calcd for $C_{76}H_{73}N_2$: 1013.5768, found: 1013.5767.

Synthesis of the 1,3-bis(2,6-bis(di-*p*-tolylmethyl)-4-methylphenyl)imidazolium chloride, IPr^{*Tol}·HCl (**5**):

Diazabutadiene **C** (7.34 g, 7.24 mmol) dissolved in chloroform (50 mL) at 60 °C was treated with paraformaldehyde (261 mg, 8.69 mmol) and zinc chloride (1.185 g,

8.69 mmol) as a solution in HCl 36% (1.5 mL), for 1.5 h. The reaction mixture was then diluted with CH_2Cl_2 (50 mL), and washed with HCl 2 M (50 mL) and brine. The organic layer was subsequently dried over $MgSO_4$, filtered and concentrated to dryness. The resulting residue was finally suspended in Et_2O, filtered and washed thoroughly with Et_2O (3 × 15 mL) to afford **5** as a colourless powder (4.96 g, 65%)

^{1}H NMR ($CDCl_3$, 300 MHz) δ 2.18 (s, 6H, C$H_{3\text{-Tol}}$), 2.28 (s, 24H, C$H_{3\text{-Tol}}$), 5.16 (s, 4H, C*H*), 5.64 (s, 2H, H_{imid}), 6.65 (d, *J* 7.8 Hz, 8H, H_{Ar}), 6.76 (s, 4H, H_{Ar}), 6.90 (d, *J* 7.7 Hz, 8H, H_{Ar}), 7.05 (s, 16H, H_{Ar}), 12.68 (br, 1H, H_{imid}).

^{13}C NMR ($CDCl_3$, 75 MHz) δ 21.2 (*C*H_3), 22.0 (*C*H_3), 50.7 (*C*H), 123.7 (*C*H_{Imid}), 129.2 (*C*H_{Ar}), 129.3 (*C*H_{Ar}), 129.4 (*C*H_{Ar}), 130.0 (*C*H_{Ar}), 130.7 (*C*H_{Ar}), 136.2 (C_{Ar}), 136.4 (C_{Ar}), 139.4 (C_{Ar}), 139.6 (C_{Ar}), 140.9 (C_{Ar}), 141.3 (C_{Ar}).

HRMS $(NESI)^+$: Calcd for $C_{77}H_{73}N_2$ 1025.5768, found: 1025.5748.

Synthesis of the [Pd(IPr*^{Tol})(cinnamyl)Cl] (**4**):

In a glovebox, a 500 mL round-bottomed flask equipped with a magnetic stirring bar was charged with IPr*^{Tol}•HCl (**5**) (2.00 g, 1.88 mmol) and 150 mL of THF. KO*t*-Bu (230 mg, 2.05 mmol) was then added as a powder and the reacting media was stirred at room temperature for 4 h. [{Pd(cinnamyl)(μ-Cl)}$_2$] (443 mg, 0.85 mmol) was subsequently added, as a solution in THF, and stirring was continued overnight outside of the glovebox. The solvent was eventually evaporated off, and the crude residue was dissolved into CH_2Cl_2 (15 mL) before being passed through a bed of Celite® over silica (2 cm of each). The collected filtrate was concentrated to dryness and further dried under high vacuum. Complex **5** was finally recovered as a pale yellow powder (2.11 g, 97%).

^{1}H NMR (CD_2Cl_2, 400 MHz) δ 1.15 (d, *J* 12.0 Hz, 1H, H_{cin}) 2.25–2.32 (m, 30H, CH_3), 2.54 (d, *J* 6.5 Hz, 1H, H_{cin}), 4.48 (d, *J* 12.6 Hz, 1H, H_{cin}), 4.92–4.96 (m, 1H, H_{cin}), 5.41 (s, 2H, H_{imid}), 5.70 (s, 2H, C*H*), 5.86 (s, 2H, C*H*), 6.69–6.73 (m, 8H, H_{Ar}), 6.90–6.94 (m, 12H, H_{Ar}), 7.03–7.09 (m, 8H, H_{Ar}), 7.15–7.21 (m, 8H, H_{Ar}), 7.36–7.46 (m, 5H, H_{Ar}).

^{13}C NMR (CD_2Cl_2, 75 MHz) δ 21.2 (*C*H_3), 21.3 (*C*H_3), 22.0 (*C*H_3), 47.8 (*C*$H_{2\text{-}Cin}$), 51.2 (*C*H), 91.0 (*C*H_{Cin}), 109.4 (*C*H_{Cin}), 123.9 (*C*H_{imid}), 127.5 (*C*H_{Ar}), 128.0 (*C*H_{Ar}), 128.9 (*C*H_{Ar}), 129.2 (*C*H_{Ar}), 129.5 (*C*H_{Ar}), 130.3 (*C*H_{Ar}), 130.9 (*C*H_{Ar}), 136.3 (C_{Ar}), 138.6 (C_{Ar}), 138.7 (C_{Ar}), 141.4 (C_{Ar}), 141.5 (C_{Ar}), 141.6 (C_{Ar}), 142.0 (C_{Ar}), 142.4 (C_{Ar}), 142.5 (C_{Ar}), 183.1 (C_{Ar}).

Elemental Analysis: Calcd for $C_{86}H_{81}N_2ClPd$, C (80.42), H (6.36), N (2.18); found C (80.54), H (6.48), N (2.24).

X-Ray: CCDC 887349 (See Supporting Information Files 2 and 3).

Optimisation reactions:

Optimisation of the solvent/ base combination[a]

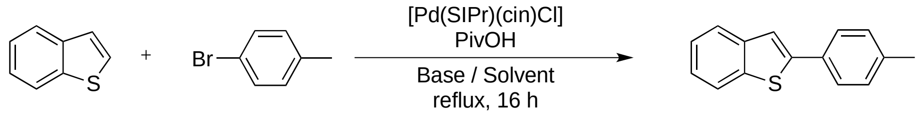

Solvent	Base	Conversion (%)[b]
Dioxane	LiHMDS	74
Dioxane	Cs_2CO_3	18
Dioxane	KOH	0
Dioxane	KO*t*-Bu	0
Dioxane	NaO*t*-Bu	77
Toluene	LiHMDS	31
Toluene	Cs_2CO_3	0
Toluene	KOH	0
Toluene	KO*t*-Bu	9
Toluene	NaO*t*-Bu	34
DMF	LiHMDS	0
DMF	Cs_2CO_3	>99
DMF	KOH	0
DMF	KO*t*-Bu	0
DMF	NaO*t*-Bu	11
DMF	KOAc	96
DMA	LiHMDS	8
DMA	KOH	27
DMA	KO*t*-Bu	48
DMA	NaO*t*-Bu	44
DMA	Cs_2CO_3	>99
DMA	KOAc	91
DMA	Cs_2CO_3	25[c]
DMA	K_2CO_3	>99[c]
DMA	Na_2CO_3	59[c]

[a]Reaction conditions: benzothiophene (0.6 mmol), 4-bromotoluene (1.2 mmol), base (0.9 mmol), pivalic acid (30 mol %), [Pd(SIPr)(cin)Cl] (2 mol %), solvent (2 mL).

[b]Conversion to C–H arylated product determined by GC.

[c][Pd(SIPr)(cin)Cl] (1 mol %)

Optimisation of the catalyst loading[a]

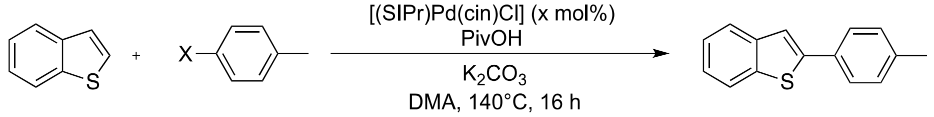

Catalyst loading	X	Conversion (%)[b]
2 mol %	Br	>99
1 mol %	Br	>99
0.5 mol %	Br	>99
0.1 mol %	Br	>99
0.1 mol %	Cl	0
0.1 mol %	I	45
0.05 mol %	Br	92
0.025 mol %	Br	80 (50)[c]
0.01 mol %	Br	35

[a]Reaction conditions: benzothiophene (0.6 mmol), 4-bromotoluene (0.6 mmol), K_2CO_3 (0.9 mmol), pivalic acid (30 mol %), [Pd(SIPr)(cin)Cl] (x mol %), DMA (2 mL).

[b]Conversion to C–H arylated product determined by GC.

[c]Yield in parenthesis refers to a reaction performed in DMF.

Additives screening[a]

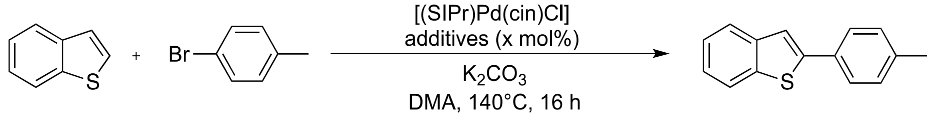

Additives	Additive loading	Conversion (%)[b]
CF_3COOH	30 mol %	<1
CH_3COOH	30 mol %	5
PhCOOH	30 mol %	12
PivOH	30 mol %	75
PivOH	20 mol %	66
PivOH	10 mol %	55

[a]Reaction conditions: benzothiophene (0.6 mmol), 4-bromotoluene (0.6 mmol), K_2CO_3 (0.9 mmol), additives (x mol %), [Pd(SIPr)(cin)Cl] (0.025 mol %), DMA (2 mL).

[b]Conversion to C–H arylated product determined by GC.

[c]Yield in parenthesis refers to a reaction performed in DMF.

General procedure for the direct arylation of heterocycles

In a glovebox, a vial containing a stirring bar was charged with K_2CO_3 (124 mg, 0.9 mmol, 1.5 equiv) and pivalic acid (0.18 mmol, 18 mg, 30 mol %), and sealed with a screw cap fitted with a septum. The heterocycle (0.6 mmol, 1.0 equiv) and/or the arylbromide (0.6 mmol, 1.0 equiv) were added at this point if in solid form, and DMA (1.9 mL) was poured into the vial. Outside of the glovebox, the heterocycle and/or the aryl bromide were added at this point if in liquid form. Finally, [Pd(SIPr)(cin)Cl] (**1**) was added as a 0.06 M solution in DMA (0.6–6 µmol, 10–100 µL, 0.01–0.1 mol %), and the vial was heated to 140 °C for 16 h. The solution was then cooled to room temperature, diluted with 40 mL of ethyl acetate and washed with water (2 × 20 mL) and brine (20 mL). The organic layer was dried over $MgSO_4$, filtered and concentrated in vacuo. The crude residue was finally purified by either trituration in pentane (if not soluble) or by silica gel column chromatography.

Characterisation data of compounds 8a–8g, 10a–10c, 12a–12c and 14a–14c:

2-(*p*-tolyl)benzo[*b*]thiophene (**8a**) [2]

The general procedure yielded, after trituration in pentane, 120 mg (89%) of the title compound as an off-white solid.

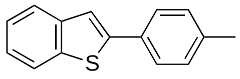

^{1}H NMR (CD_2Cl_2, 300 MHz) δ 2.40 (s, 3H, CH_3), 7.25–7.39 (m, 4H, H_{Ar}), 7.54 (s, 1H, H_{Ar}), 7.62–7.65 (m, 2H, H_{Ar}), 7.76–7.86 (m, 2H, H_{Ar}).

^{13}C NMR (CD_2Cl_2, 100 MHz) δ 21.5, 119.4, 122.7, 124.0, 124.7, 125.1, 126.8, 130.2, 131.9, 139.1, 139.8, 141.4, 144.9.

2-(*m*-tolyl)benzo[*b*]thiophene (8b) [3]:

The general procedure yielded, after flash chromatography on silica gel (pentane), 108 mg (80%) of the title compound as a colourless solid.

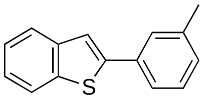

^{1}H NMR (CD_2Cl_2, 400 MHz) δ 2.44 (s, 3H, CH_3), 7.21 (br d, *J* 7.5 Hz, 1H, H_{Ar}), 7.32–7.41 (m, 3H, H_{Ar}), 7.56 (br d, *J* 7.9 Hz, 1H, H_{Ar}), 7.80–7.82 (m, 1H, H_{Ar}), 7.85–7.88 (m, 1H, H_{Ar}).

^{13}C NMR (CD_2Cl_2, 100 MHz) δ 21.7, 119.9, 122.8, 124.0, 124.1, 124.9, 125.1, 127.6, 129.4, 129.7, 134.6, 139.4, 139.9, 141.3, 144.9.

2-(*o*-tolyl)benzo[*b*]thiophene (**8c**) [4]:

The general procedure yielded, after trituration in pentane, 104 mg (77%) of the title compound as a colourless solid.

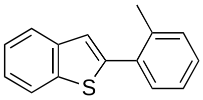

^{1}H NMR (CD_2Cl_2, 300 MHz) δ 2.49 (s, 3H, CH_3), 7.25–7.43 (m, 6H, H_{Ar}), 7.48–7.51 (m, 1H, H_{Ar}), 7.81–7.84 (m, 1H, H_{Ar}), 7.80–7.82 (m, 1H, H_{Ar}), 7.86–7.89 (m, 1H, H_{Ar}).

^{13}C NMR (CD_2Cl_2, 100 MHz) δ 21.4, 122.5, 123.6, 124.0, 124.7, 124.9 126.5, 128.9, 131.1, 131.4, 134.6, 137.0, 140.6, 140.8, 144.0.

2-(4-methoxyphenyl)benzo[*b*]thiophene (**8d**) [2]:

The general procedure yielded, after trituration in pentane, 101 mg (70%) of the title compound as a colourless solid.

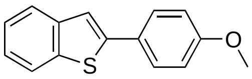

^{1}H NMR (CD_2Cl_2, 400 MHz) δ 3.85 (s, 3H, CH_3), 6.96–6.98 (m, 2H, H_{Ar}), 7.29 (ddd, *J* 7.8, 7.1 and 1.4 Hz, 1H, H_{Ar}), 7.34 (ddd, *J* 7.8, 7.1 and 1.3 Hz, 1H, H_{Ar}), 7.46 (d, *J* 0.8 Hz, 1H, H_{Ar}), 7.64–7.68 (m, 2H, H_{Ar}), 7.75–7.77 (m, 1H, H_{Ar}), 7.81–7.83 (m, 1H, H_{Ar}).

^{13}C NMR (CD_2Cl_2, 100 MHz) δ 55.9, 114.9, 118.7, 122.7, 123.8, 124.5, 125.1, 127.4, 128.2, 139.6, 141.5, 144.6, 160.5.

2-(4-chlorophenyl)benzo[*b*]thiophene (**8e**) [2]:

The general procedure yielded, after trituration in pentane, 72 mg (49%) of the title compound as an off-white solid.

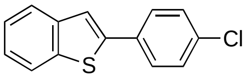

^{1}H NMR (CD_2Cl_2, 300 MHz) δ 7.33–7.38 (m, 2H, H_{Ar}), 7.39–7.44 (m, 2H, H_{Ar}), 7.57 (s, 1H, H_{Ar}), 7.66–7.69 (m, 2H, H_{Ar}), 7.78–7.81 (m, 1H, H_{Ar}), 7.83–7.86 (m, 1H, H_{Ar}).

^{13}C NMR (CD_2Cl_2, 75 MHz) δ 120.5, 122.8, 124.2, 125.2, 125.3, 128.2, 129.6, 133.4, 134.5, 140.0, 141.2, 143.2.

2-(4-fluorophenyl)benzo[*b*]thiophene (**8f**) [2]:

The general procedure yielded, after trituration in pentane, 73 mg (53%) of the title compound as an off-white solid.

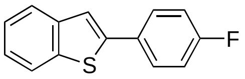

^{1}H NMR (CD_2Cl_2, 300 MHz) δ 7.12–7.18 (m, 2H, H_{Ar}), 7.30–7.40 (m, 2H, H_{Ar}), 7.52 (s, 1H, H_{Ar}), 7.69–7.74 (m, 2H, H_{Ar}), 7.78–7.86 (m, 2H, H_{Ar}).

^{13}C NMR (CD_2Cl_2, 75 MHz) δ 116.5 (d, J_{CF} 22 Hz), 120.4, 122.7, 124.1, 125.0, 125.2, 128.7 (d, J_{CF} 8 Hz), 131.1 (d, J_{CF} 3 Hz), 139.9, 141.3, 143.5, 163.4 (d, J_{CF} 248 Hz).

4-(benzo[*b*]thiophen-2-yl)benzaldehyde (**8g**) [5]:

The general procedure yielded, after flash chromatography on silica gel (pentane), 53 mg (37%) of the title compound as a colourless solid.

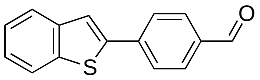

^{1}H NMR (CD_2Cl_2, 300 MHz) δ 7.34–7.42 (m, 2H, H_{Ar}), 7.70 (s, 1H, H_{Ar}), 7.80–7.91 (m, 6H, H_{Ar}), 10.00 (s, 1H, C*H*O).

^{13}C NMR (CD_2Cl_2, 75 MHz) δ 122.2, 122.9, 124.6, 125.4, 125.7, 127.1, 130.7, 136.3, 140.2, 140.7, 141.0, 142.9, 191.8.

HRMS (APCI)$^+$: Calcd for $C_{15}H_{11}OS$ 239.0525, found 239.0527.

2-(*p*-tolyl)-3-methylbenzo[*b*]thiophene (**10a**) [6]:

The general procedure yielded, after trituration in pentane, 121 mg (85%) of the title compound as an off-white solid.

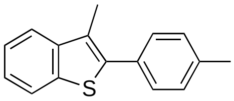

^{1}H NMR (CD_2Cl_2, 300 MHz) δ 2.42 (s, 3H, C*H*$_3$), 2.46 (s, 3H, C*H*$_3$), 7.28–7.47 (m, 6H, H_{Ar}), 7.74 (dt, J 8.4 and 1.0 Hz, 1H, H_{Ar}), 7.82–7.85 (m, 1H, H_{Ar}).

^{13}C NMR (CD_2Cl_2, 75 MHz) δ 13.0, 22.5, 122.5, 122.6, 124.6, 124.7, 127.7, 129.8, 130.0, 132.2, 138.5, 138.6, 139.2, 141.9.

2-(*o*-tolyl)-3-methylbenzo[*b*]thiophene (**10b**):

The general procedure yielded, after flash chromatography on silica gel, 119 mg (83%) of the title compound as a colourless solid.

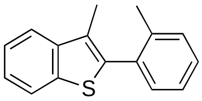

^{1}H NMR (CD_2Cl_2, 300 MHz) δ 2.21 (s, 3H, CH_3), 2.25 (s, 3H, CH_3), 7.24–7.30 (m, 1H, H_{Ar}), 7.32–7.37 (m, 4H, H_{Ar}), 7.44 (ddd, *J* 7.9, 7.1 and 1.3 Hz, 1H, H_{Ar}), 7.75 (ddd, J 7.9, 1.4 and 0.8 Hz, 1H, H_{Ar}), 7.85 (ddd, J 7.8, 1.4 and 0.7 Hz, 1H, H_{Ar}).

^{13}C NMR (CD_2Cl_2, 75 MHz) δ 13.0, 21.5, 122.5, 122.6, 124.6, 124.7, 127.7, 129.8, 130.0, 132.2, 138.5, 138.6, 139.2, 141.9.

HRMS (APCI)$^+$: Calcd for $C_{16}H_{15}S$ 239.0889, found 239.0891.

2-(4-fluorophenyl)-3-methylbenzo[*b*]thiophene (**10c**):

The general procedure yielded, after trituration in pentane, 76 mg (52%) of the title compound as an off-white solid.

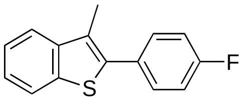

^{1}H NMR (CD_2Cl_2, 300 MHz) δ 2.46 (s, 3H, CH_3), 7.16–7.22 (m, 2H, H_{Ar}), 7.38–7.45 (m, 2H, H_{Ar}), 7.53–7.57 (m, 2H, H_{Ar}), 7.74–7.78 (m, 1H, H_{Ar}), 7.84–7.87 (m, 1H, H_{Ar}).

^{13}C NMR (CD_2Cl_2, 75 MHz) δ 12.9, 116.0 (d, J_{CF} 22 Hz), 122.6, 122.7, 125.0, 128.3, 131.4 (d, J_{CF} 3 Hz), 132.0 (d, J_{CF} 8 Hz), 137.3, 139.3, 141.7, 163.0 (d, J_{CF} 247 Hz).

HRMS (APCI)$^+$: Calcd for $C_{15}H_{11}SF$ 242.0560, found 242.0564.

2-(*p*-tolyl)-5-methylthiophene (**12a**) [7]:

The general procedure yielded, after flash chromatography on silica gel (pentane), 102 mg (90%) of the title compound as a colourless solid.

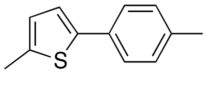

^{1}H NMR (CD_2Cl_2, 300MHz) δ 2.35 (s, 3H, CH_3), 2.50 (s, 3H, CH_3), 6.73 (dq, *J* 3.4 and 1.1 Hz, 1H, H_{Ar}), 7.08 (d, *J* 3.5 Hz, 1H, H_{Ar}), 7.16–7.19 (m, 2H, H_{Ar}), 7.43–7.46 (m, 2H, H_{Ar}).

^{13}C NMR (CD_2Cl_2, 75MHz) δ 15.7, 21.4, 122.9, 125.7, 126.7, 130.0, 132.4, 137.5, 139.6, 142.5.

2-(4-methoxyphenyl)-5-methylthiophene (**12b**) [8]:

The general procedure yielded, after flash chromatography on silica gel (pentane), 92 mg (75%) of the title compound as a colourless solid.

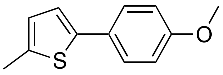

^{1}H NMR (CD_2Cl_2, 400 MHz) δ 2.48 (d, *J* 1.1 Hz, 3H, CH_3), 3.81 (s, 3H, CH_3), 6.71 (dq, *J* 3.5 and 1.2 Hz, 1H, H_{Ar}), 6.87–6.91 (m, 2H, H_{Ar}), 7.00 (d, *J* 3.5 Hz, 1H, H_{Ar}), 7.46–7.49 (m, 2H, H_{Ar}).

^{13}C NMR (CD_2Cl_2, 75 MHz) δ 15.7, 55.8, 114.7, 122.3, 126.7, 127.1, 128.0, 139.1, 142.3, 159.5.

2-(4-fluorophenyl)-5-methylthiophene (**12c**) [9]:

The general procedure yielded, after flash chromatography on silica gel (pentane), 66 mg (57%) of the title compound as a colourless solid.

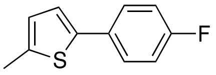

^{1}H NMR (CD_2Cl_2, 400 MHz) δ 2.51 (s, 3H, CH_3), 6.75 (dq, *J* 3.5 and 1.1 Hz, 1H, H_{Ar}), 7.05–7.11 (m, 3H, H_{Ar}), 7.54 (ddt, *J* 7.1, 5.2 and 2.5 Hz, 2H, H_{Ar}).

^{13}C NMR (CD_2Cl_2, 75 MHz) δ 15.7, 116.2 (d, J_{CF} 22 Hz), 123.5, 126.8, 127.6 (d, J_{CF} 8 Hz), 131.6 (d, J_{CF} 3 Hz), 140.3, 141.2, 162.6 (d, J_{CF} 246 Hz).

3-(*p*-tolyl)imidazo[1,2,*a*]pyridine (**14a**) [10]:

The general procedure yielded, after flash chromatography on silica gel (DCM-MeOH: 0–2%), 92 mg or 86 mg (74% or 69%) of the title compound as a colourless solid.

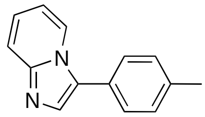

^{1}H NMR (CD_2Cl_2, 300 MHz) δ 2.43 (s, 3H, C*H*$_3$), 6.86 (tt, *J* 6.9 and 1.3 Hz, 1H, H_{Ar}), 7.18 (ddt, *J* 9.1, 6.6 and 1.3 Hz, 1H, H_{Ar}), 7.34 (d, *J* 8.0 Hz, 2H, H_{Ar}), 7.47 (d, *J* 8.2 Hz, 2H, H_{Ar}), 7.60–7.63 (m, 2H, H_{Ar}), 8.34 (dt, *J* 7.0 and 1.3 Hz, 1H, H_{Ar}).

^{13}C NMR (CD_2Cl_2, 75 MHz) δ 21.6, 112.8, 118.5, 124.0, 124.4, 126.3, 127.0, 128.3, 130.4, 132.7, 138.7, 146.5.

3-(4-methoxyphenyl)imidazo[1,2,*a*]pyridine (**14b**) [11]:

The general procedure yielded, after flash chromatography on silica gel (DCM-MeOH: 0–4%), 71 mg (53%) of the title compound as a colourless solid.

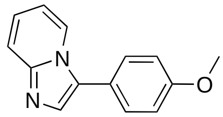

^{1}H NMR (CD_2Cl_2, 300 MHz) δ 3.85 (s, 3H, C*H*$_3$), 6.76 (td, *J* 6.8 and 1.3 Hz, 1H, H_{Ar}), 6.99–7.08 (m, 2H, H_{Ar}), 7.15 (ddd, *J* 9.1, 6.7 and 1.3 Hz, 1H, H_{Ar}), 7.39–7.51 (m, 2H, H_{Ar}), 7.57–7.63 (m, 2H, H_{Ar}), 8.26 (dt, *J* 7.0 and 1.2 Hz, 1H, H_{Ar}).

^{13}C NMR (CD_2Cl_2, 75 MHz) δ 21.6, 112.8, 118.5, 124.0, 124.4, 126.3, 127.0, 128.3, 130.4, 132.7, 138.7, 146.5.

3-(4-fluorophenyl)imidazo[1,2,*a*]pyridine (**14c**) [11] (76%):

The general procedure yielded, after flash chromatography on silica gel (DCM-MeOH: 0–2%), 97 mg (76%) of the title compound as a colourless solid.

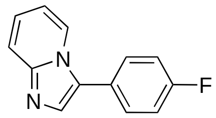

^{1}H NMR (CD_2Cl_2, 300 MHz) δ 3.85 (s, 3H, CH_3), 6.82 (td, *J* 6.8 and 1.2 Hz, 1H, H_{Ar}), 7.20–7.26 (m, 3H, H_{Ar}), 7.52–7.64 (m, 4H, H_{Ar}), 8.27 (dt, *J* 7.0 and 1.2 Hz, 1H, H_{Ar}).

^{13}C NMR (CD_2Cl_2, 75 MHz) δ 113.0, 116.7 (d, J_{CF} 22 Hz), 118.6, 123.8, 124.6, 125.2, 126.2 (d, J_{CF} 3 Hz), 130.5 (d, J_{CF} 8 Hz), 133.1, 146.6, 163.0 (d, J_{CF} 247 Hz).

References:

1. Padmanaban, M.; Biju, A. T.; Glorius, F. *Org. Lett.* **2011**, *13*, 98–101.
2. Liégault, B.; Lapointe, D.; Caron, L.; Vlassova, A.; Fagnou, K. *J. Org. Chem.* **2009**, *74*, 1826–1834.
3. Sun, L.-L.; Deng, C.-L.; Tang, R.-Y.; Zhang, X.-G. *J. Org. Chem.* **2011**, *76*, 7546–7550.
4. Bryan, C. S.; Braunger, J. A.; Lautens, M. *Angew. Chem., Int. Ed.* **2009**, *48*, 7064–7068.
5. Ohta, A.; Akita, Y.; Ohkuwa, T.; Chiba, M.; Fukunaga, R.; Miyafuji, A.; Nakata,T.; Tani, N.; Aoyagi, Y. *Heterocycles* **1990**, *31*, 1951.
6. Lange, P. P.; Gooßen, L. J.; Podmore, P.; Underwood, T.; Sciammetta, N. *Chem. Commun.* **2011**, *47*, 3628.
7. Tamba, S.; Okubo, Y.; Tanaka, S.; Monguchi, D.; Mori, A. *J. Org. Chem.* **2010**, *75*, 6998–7001.
8. Join, B.; Yamamoto, T.; Itami, K. *Angew. Chem., Int. Ed.* **2009**, *48*, 3644–3647.
9. Roger, J.; Požgan, F.; Doucet, H. *Green Chem.* **2009**, *11*, 425–432.
10. Wu, Z.; Pan, Y.; Zhou, X. *Synthesis* **2011**, *2011*, 2255–2260.
11. Fu, H. Y.; Chen, L.; Doucet, H. *J. Org. Chem.* **2012**, *77*, 4473–4478.

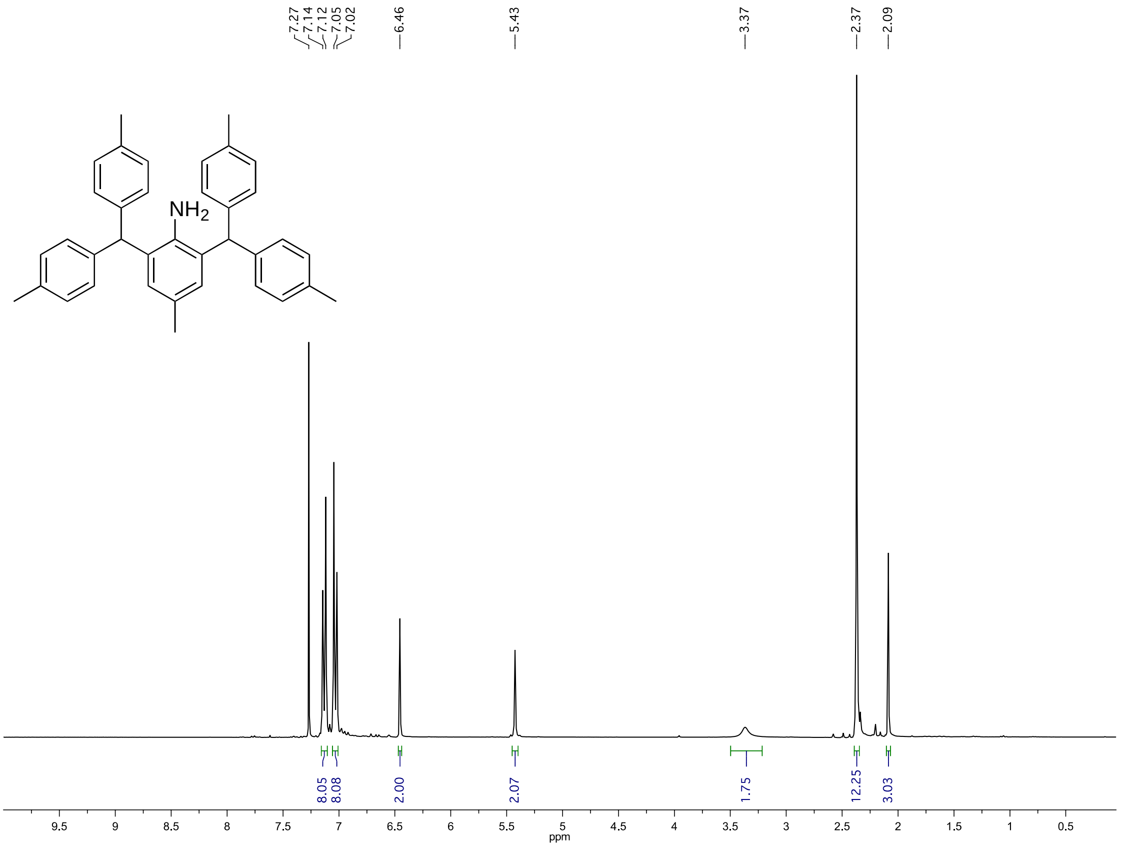
7.27
7.14
7.12
7.05
7.02
6.46
5.43
3.37
2.37
2.09
NH2
8.05
8.08
2.00
2.07
1.75
12.25
3.03
9.5 9 8.5 8 7.5 7 6.5 6 5.5 5 4.5 4 3.5 3 2.5 2 1.5 1 0.5
ppm

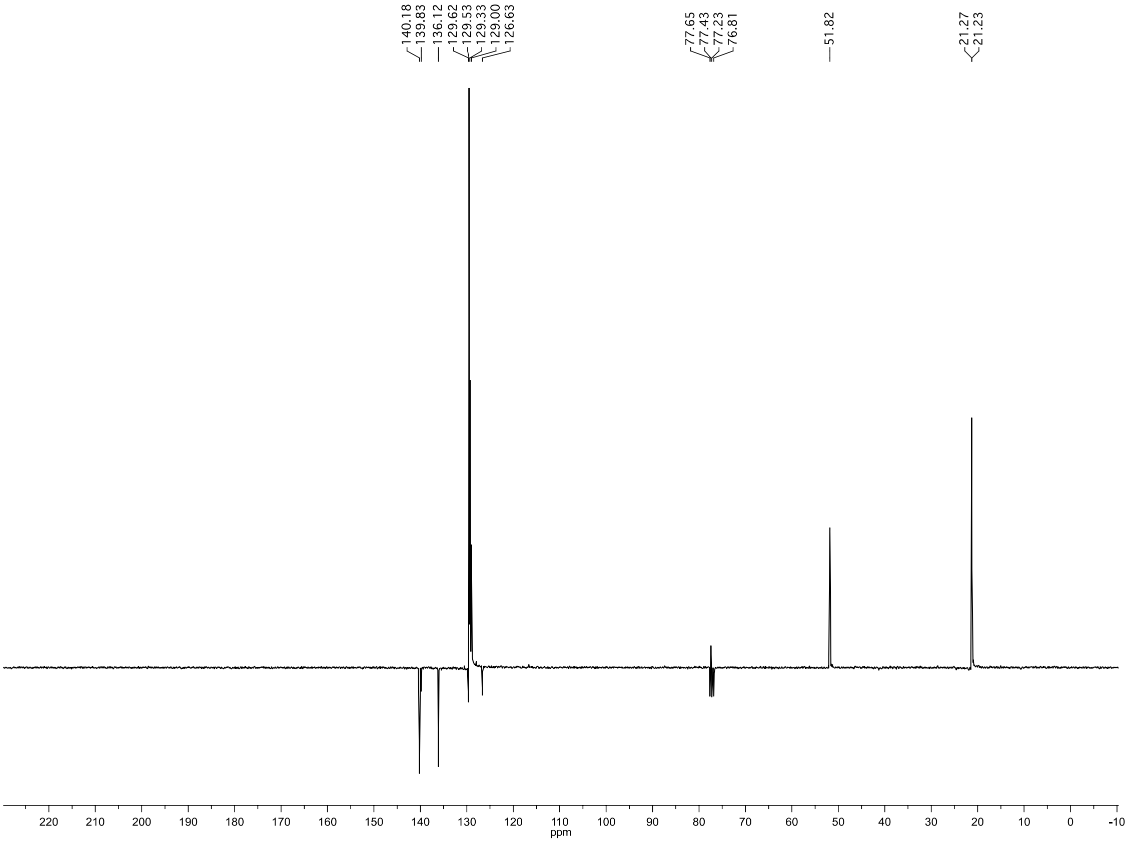
140.18
139.83
136.12
129.62
129.53
129.33
129.00
126.63
77.65
77.43
77.23
76.81
51.82
21.27
21.23
220 210 200 190 180 170 160 150 140 130 120 110 100 90 80 70 60 50 40 30 20 10 0 -10
ppm

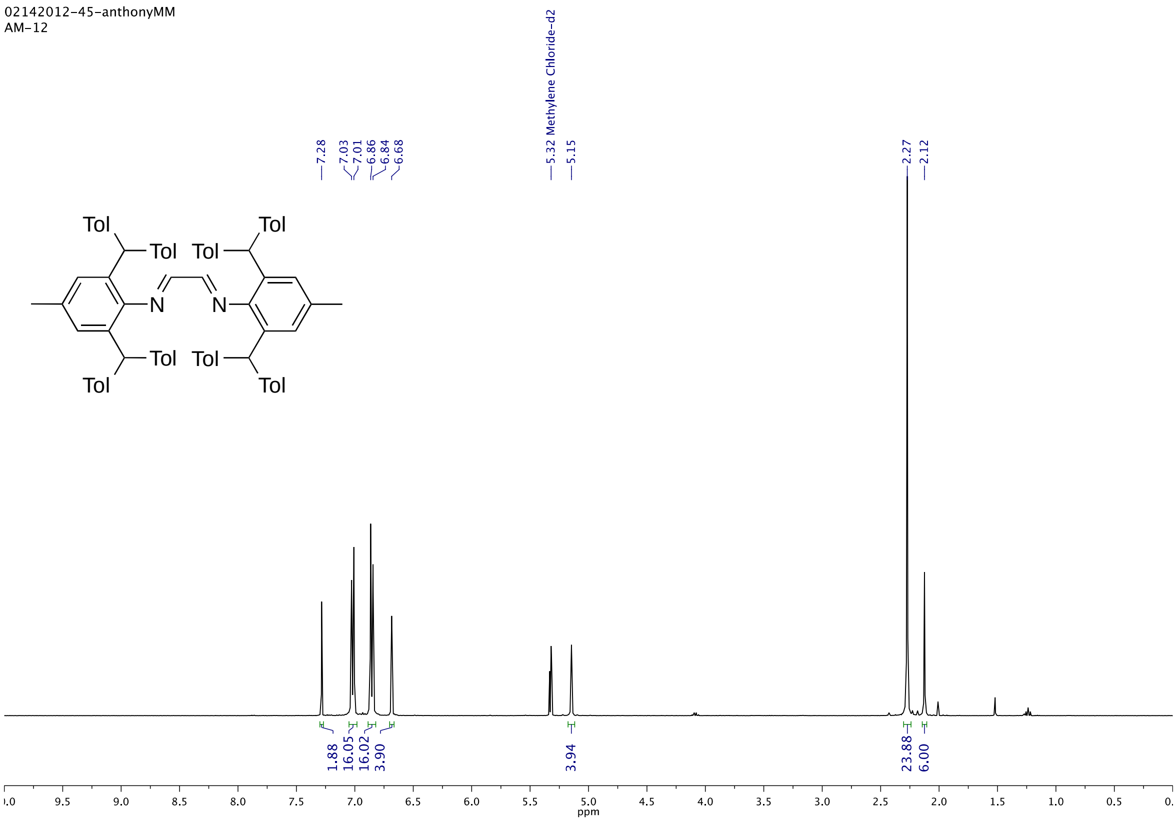
02142012-45-anthonyMM
AM-12
5.32 Methylene Chloride-d2
7.28
7.03
7.01
6.86
6.84
6.68
5.15
2.27
2.12
1.88
16.05
16.02
3.90
3.94
23.88
6.00
Tol
N
ppm

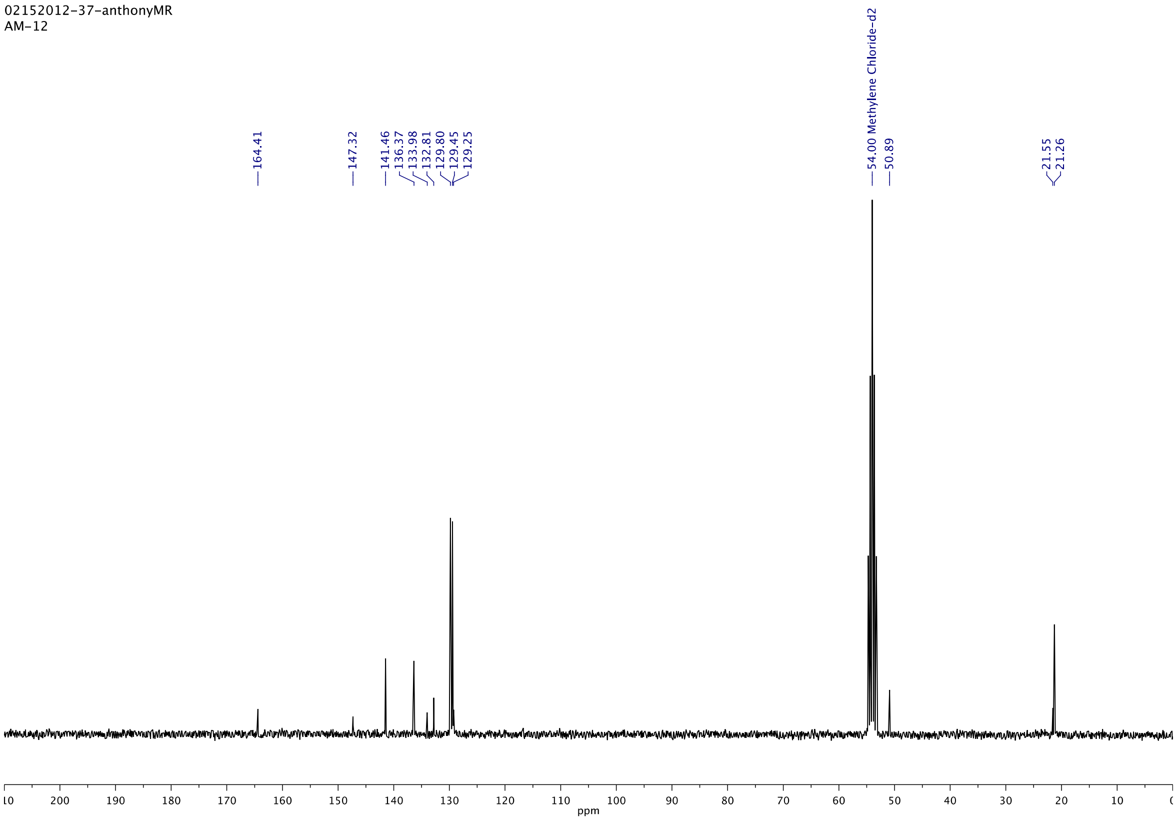
02152012-37-anthonyMR
AM-12
54.00 Methylene Chloride-d2
164.41
147.32
141.46
136.37
133.98
132.81
129.80
129.45
129.25
50.89
21.55
21.26
ppm

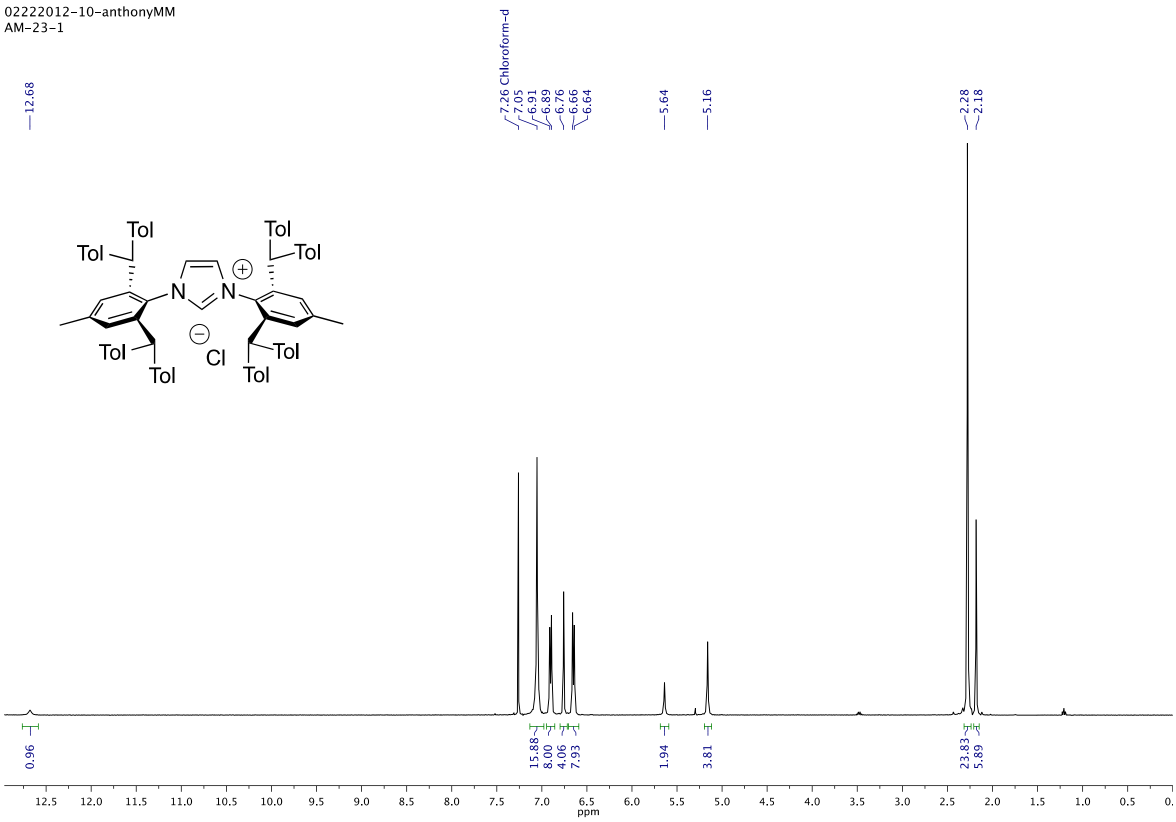
02222012-10-anthonyMM
AM-23-1
12.68
7.26 Chloroform-d
7.05
6.91
6.89
6.76
6.66
6.64
5.64
5.16
2.28
2.18
Tol
Tol
Tol
Tol
Tol
Tol
Tol
Tol
N
N
Cl
0.96
15.88
8.00
4.06
7.93
1.94
3.81
23.83
5.89
ppm

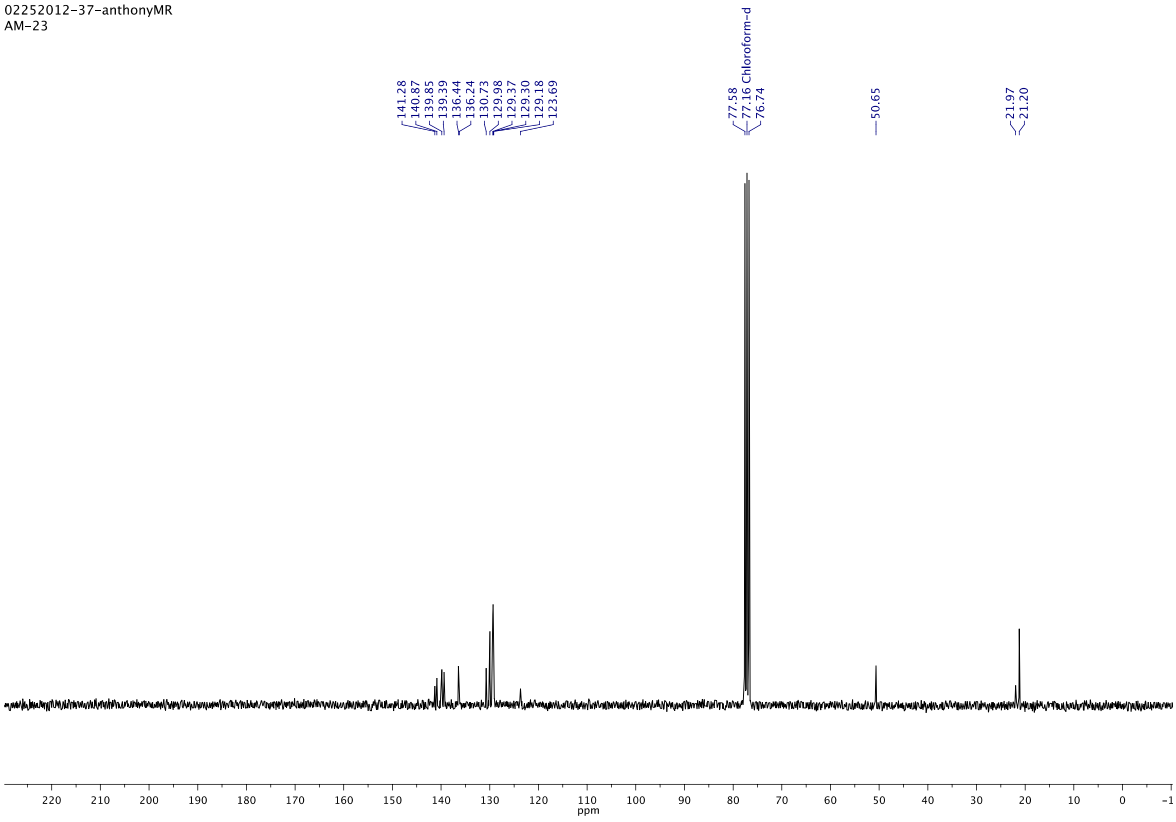
02252012-37-anthonyMR
AM-23
141.28
140.87
139.85
139.39
136.44
136.24
130.73
129.98
129.37
129.30
129.18
123.69
77.58
77.16 Chloroform-d
76.74
50.65
21.97
21.20
ppm

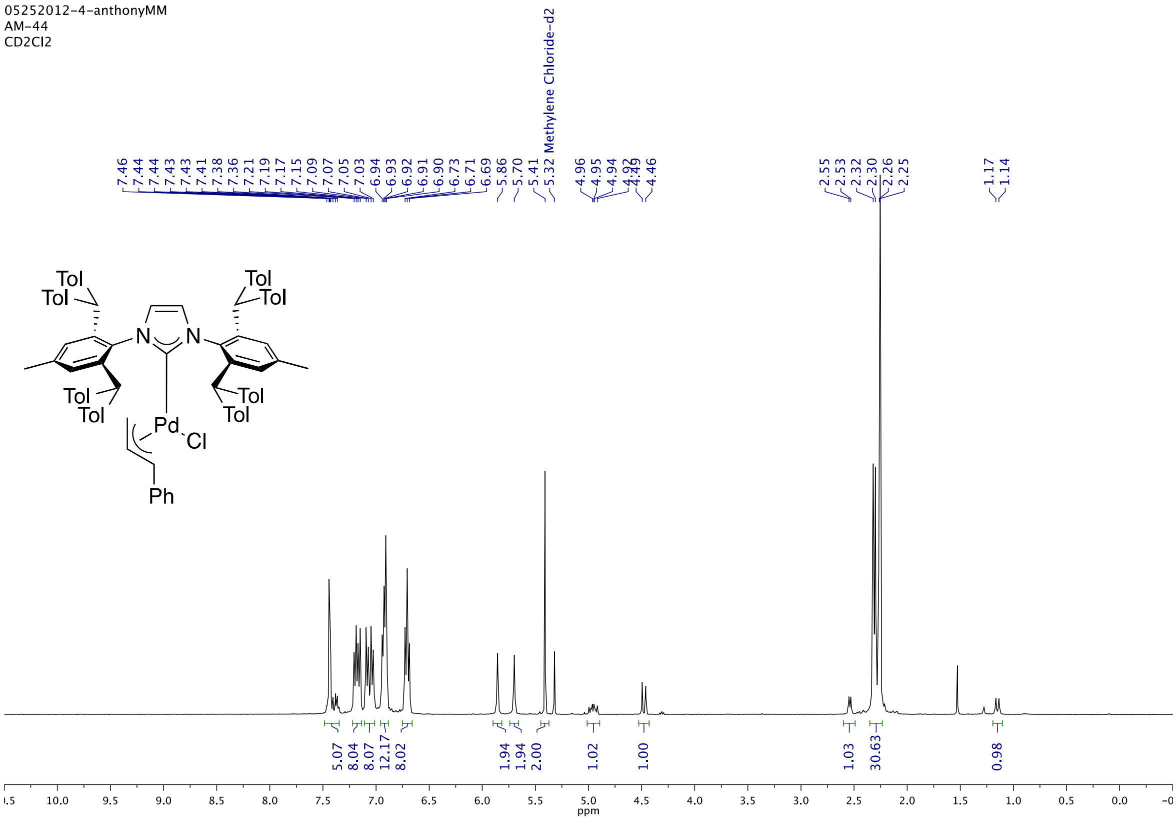
05252012-4-anthonyMM
AM-44
CD2Cl2
Methylene Chloride-d2
7.46 7.44 7.44 7.43 7.43 7.41 7.38 7.36 7.21 7.19 7.17 7.15 7.09 7.07 7.05 7.03 6.94 6.93 6.92 6.91 6.90 6.73 6.71 6.69 5.86 5.70 5.41 5.32
4.96 4.95 4.94 4.92 4.49 4.46
2.55 2.53 2.32 2.30 2.26 2.25
1.17 1.14
Tol
Tol
Tol
Tol
N
N
Tol
Tol
Tol
Tol
Pd
Cl
Ph
5.07 8.04 8.07 12.17 8.02 1.94 1.94 2.00 1.02 1.00 1.03 30.63 0.98
ppm

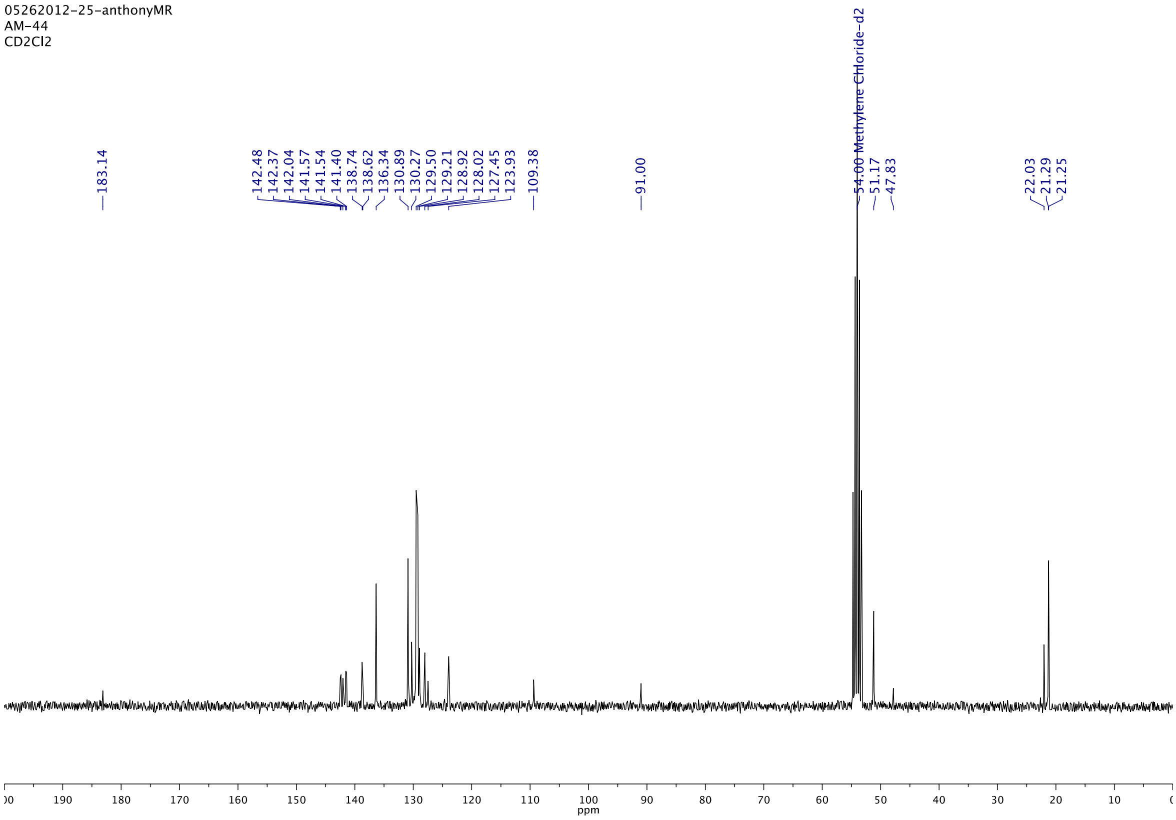
05262012-25-anthonyMR
AM-44
CD2Cl2
183.14
142.48 142.37 142.04 141.57 141.54 141.40 138.74 138.62 136.34 130.89 130.27 129.50 129.21 128.92 128.02 127.45 123.93 109.38
91.00
54.00 Methylene Chloride-d2
51.17 47.83
22.03 21.29 21.25
ppm

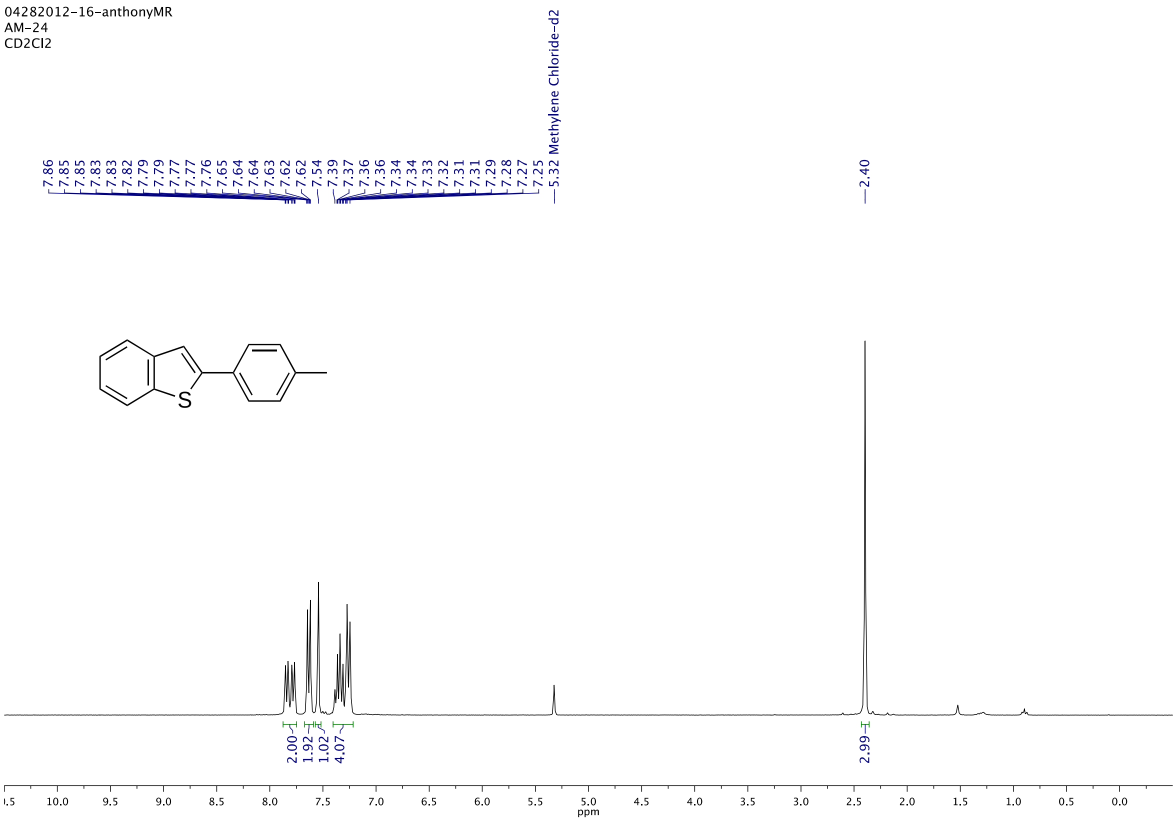
04282012-16-anthonyMR
AM-24
CD2Cl2
7.86 7.85 7.85 7.83 7.83 7.82 7.79 7.79 7.77 7.77 7.76 7.65 7.64 7.64 7.63 7.62 7.62 7.54 7.39 7.37 7.36 7.36 7.34 7.34 7.33 7.32 7.31 7.31 7.29 7.28 7.27 7.25
5.32 Methylene Chloride-d2
2.40
S
2.00
1.92
1.02
4.07
2.99
ppm

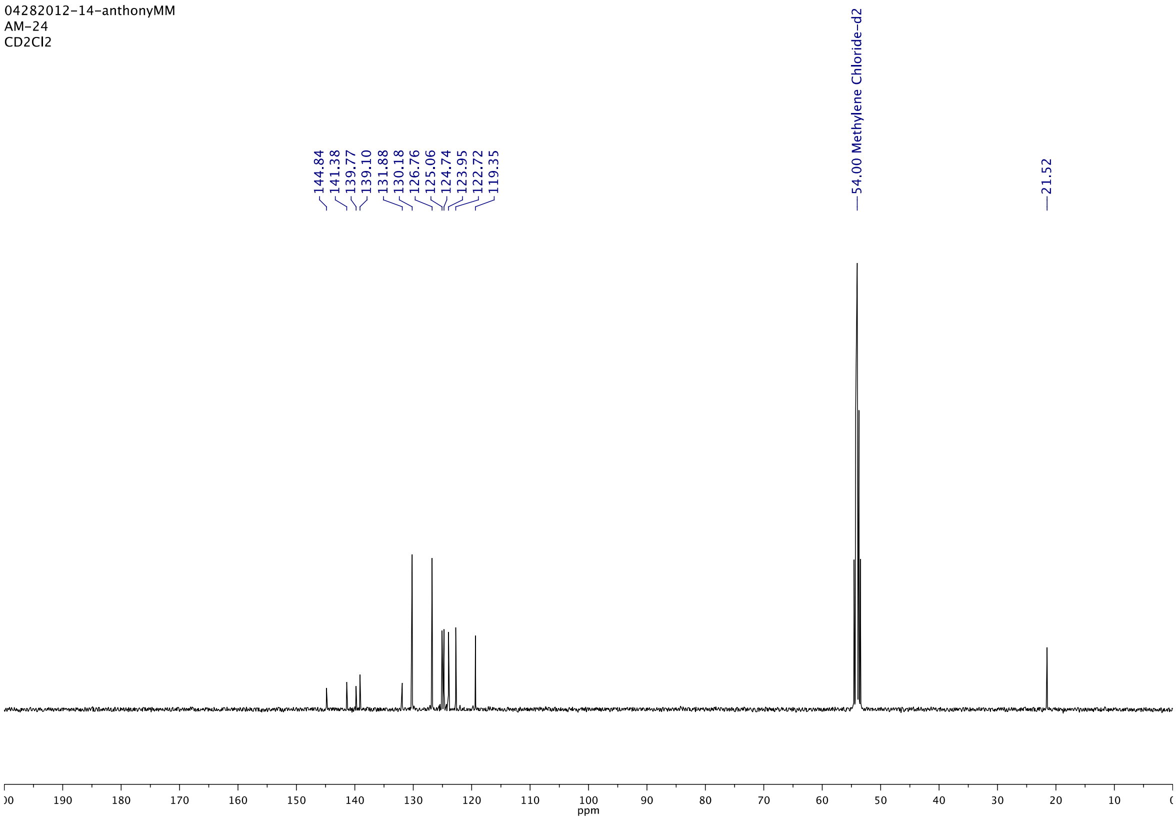
04282012-14-anthonyMM
AM-24
CD2Cl2
144.84 141.38 139.77 139.10 131.88 130.18 126.76 125.06 124.74 123.95 122.72 119.35
54.00 Methylene Chloride-d2
21.52
ppm

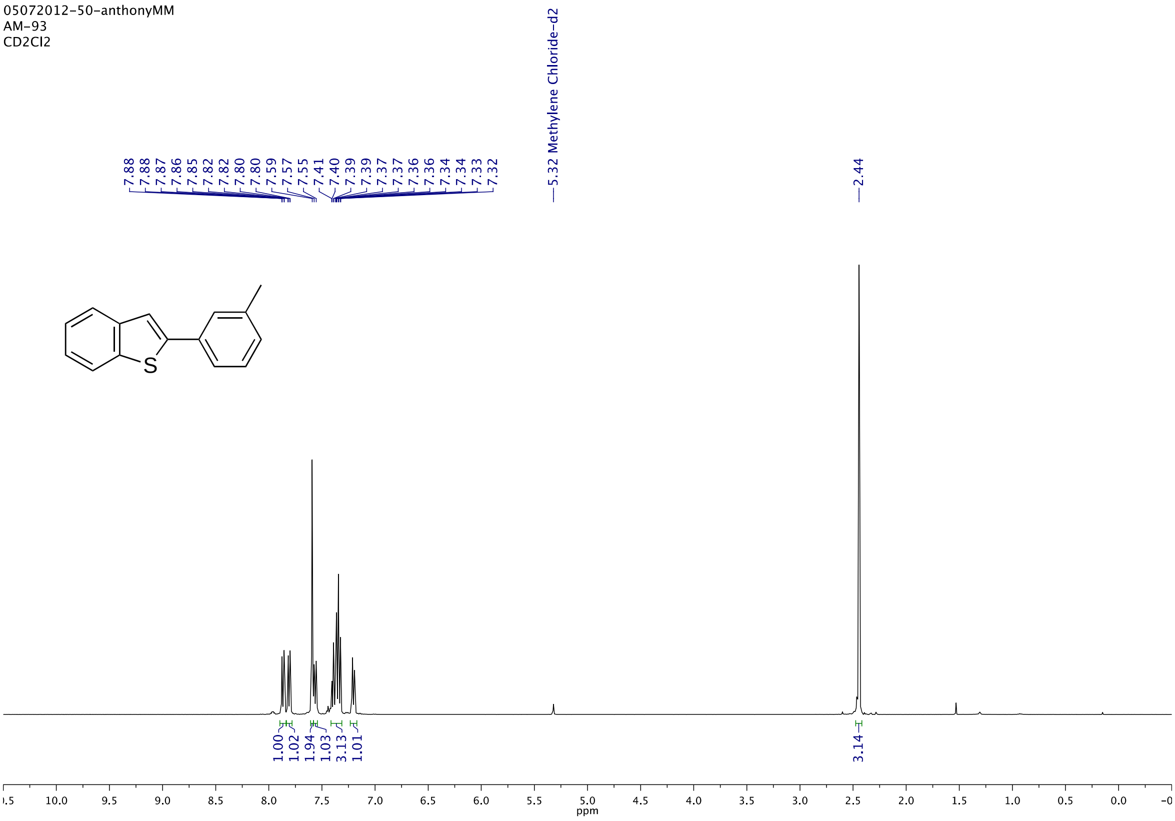
05072012-50-anthonyMM
AM-93
CD2Cl2
7.88
7.88
7.87
7.86
7.85
7.82
7.82
7.80
7.80
7.59
7.57
7.55
7.41
7.40
7.39
7.39
7.37
7.37
7.36
7.36
7.34
7.33
7.32
5.32 Methylene Chloride-d2
2.44
S
1.00
1.02
1.94
1.03
3.13
1.01
3.14
10.0
9.5
9.0
8.5
8.0
7.5
7.0
6.5
6.0
5.5
5.0
4.5
4.0
3.5
3.0
2.5
2.0
1.5
1.0
0.5
0.0
ppm

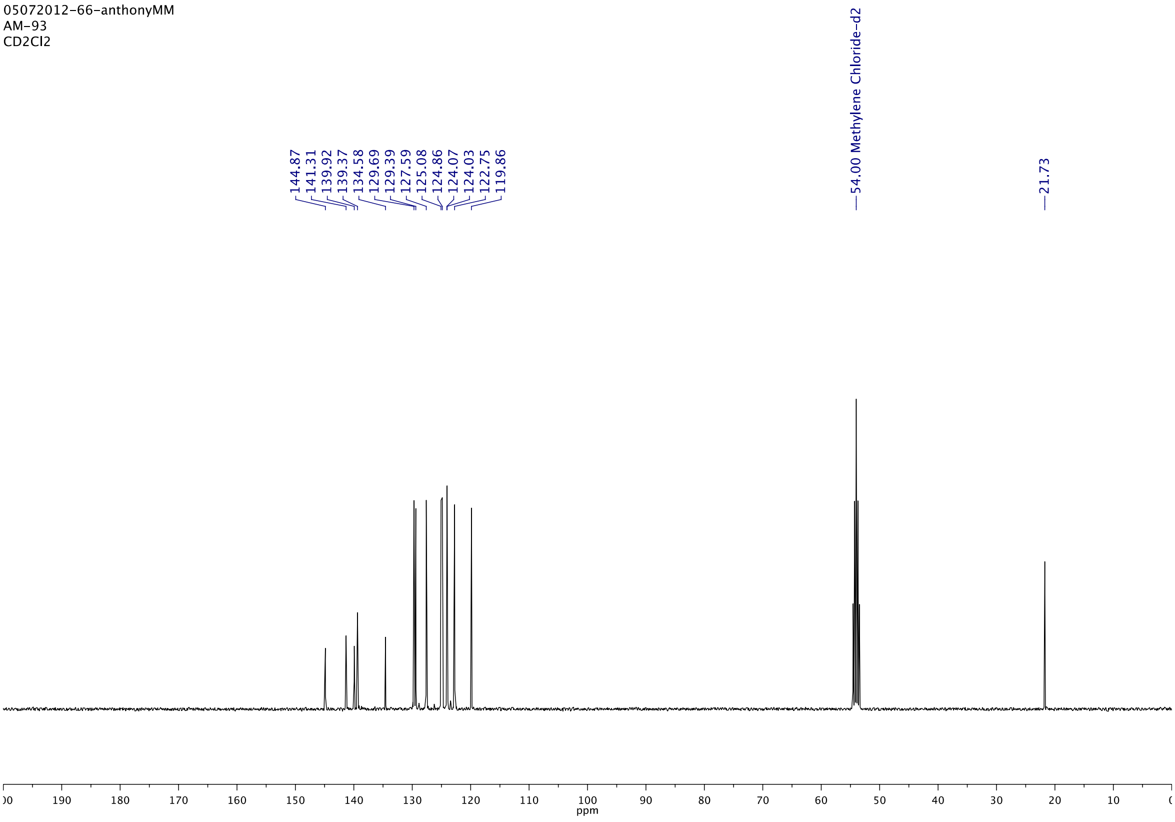
05072012-66-anthonyMM
AM-93
CD2Cl2
144.87
141.31
139.92
139.37
134.58
129.69
129.39
127.59
125.08
124.86
124.07
124.03
122.75
119.86
54.00 Methylene Chloride-d2
21.73
190
180
170
160
150
140
130
120
110
100
90
80
70
60
50
40
30
20
10
ppm

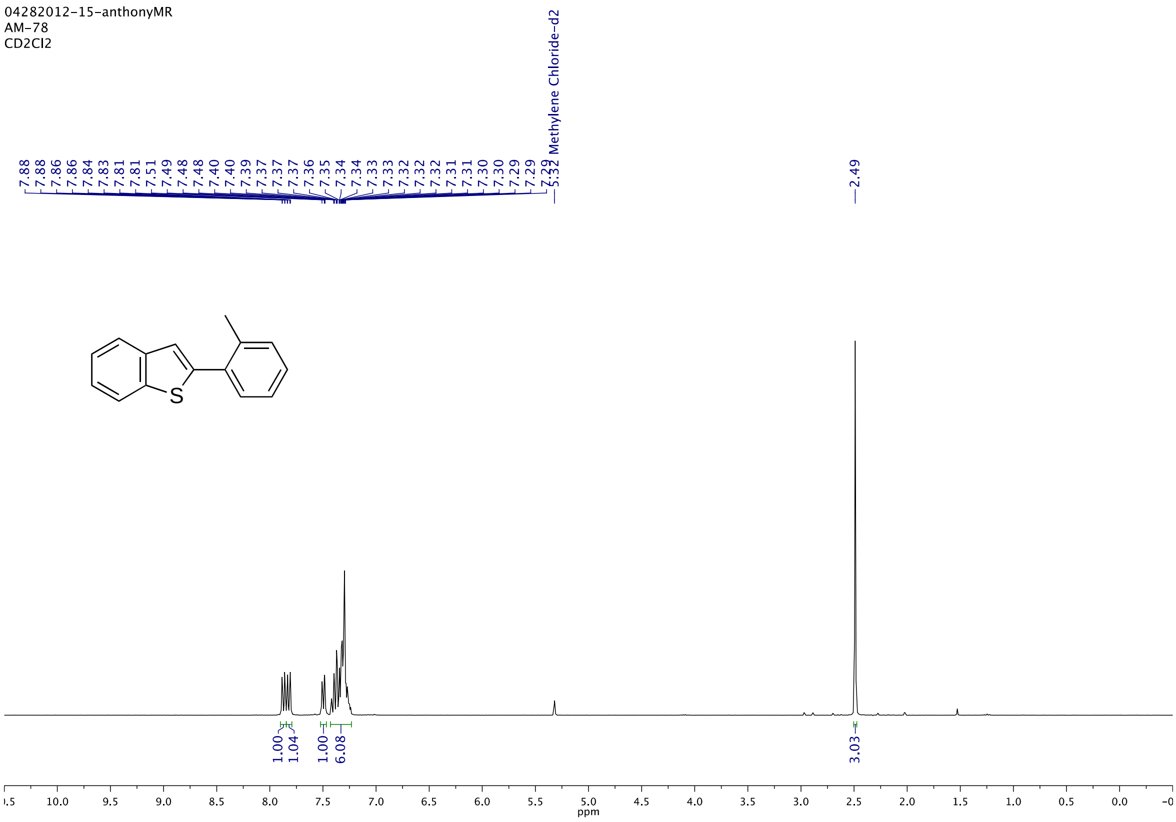
04282012-15-anthonyMR
AM-78
CD2Cl2
7.88 7.88 7.86 7.84 7.83 7.81 7.51 7.49 7.48 7.48 7.40 7.39 7.37 7.37 7.37 7.36 7.35 7.34 7.34 7.33 7.33 7.32 7.32 7.32 7.31 7.30 7.29 7.29 7.29
5.32 Methylene Chloride-d2
2.49
S
1.00 1.04 1.00 6.08 3.03
ppm

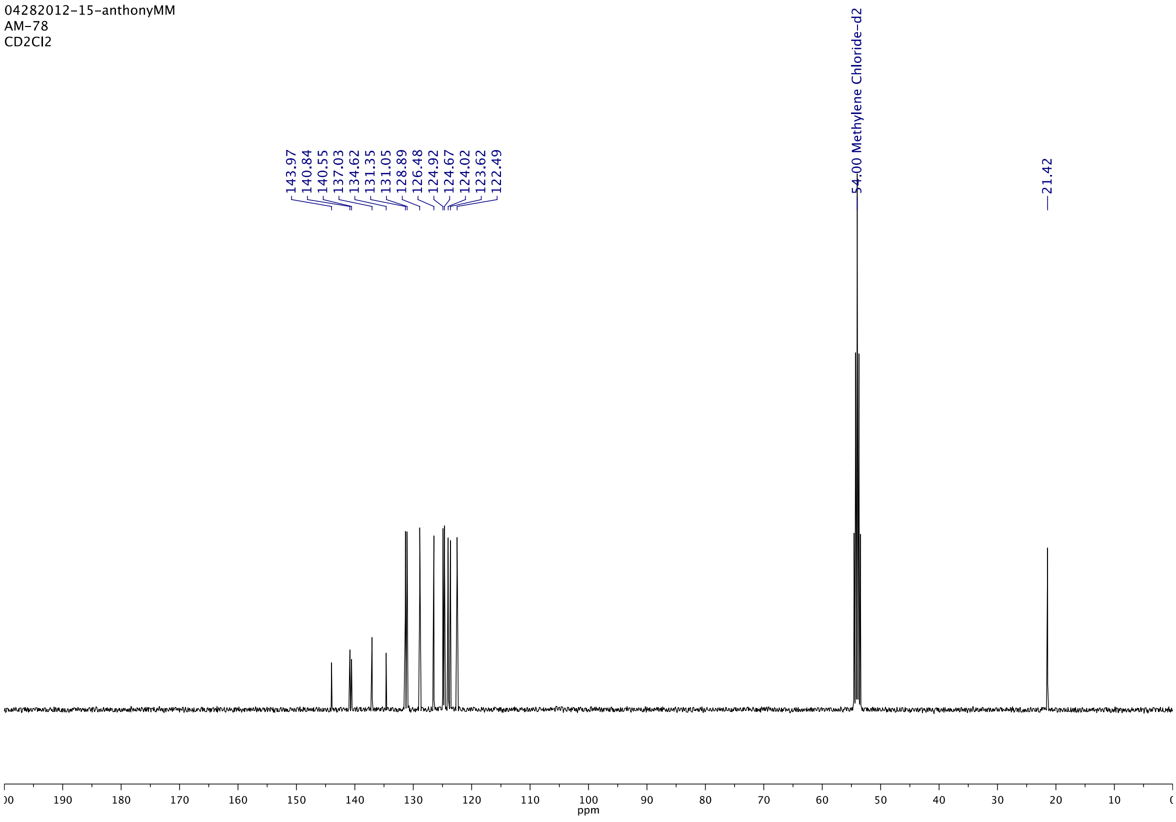
04282012-15-anthonyMM
AM-78
CD2Cl2
143.97 140.84 140.55 137.03 134.62 131.35 131.05 128.89 126.48 124.92 124.67 124.02 123.62 122.49
54.00 Methylene Chloride-d2
21.42
ppm

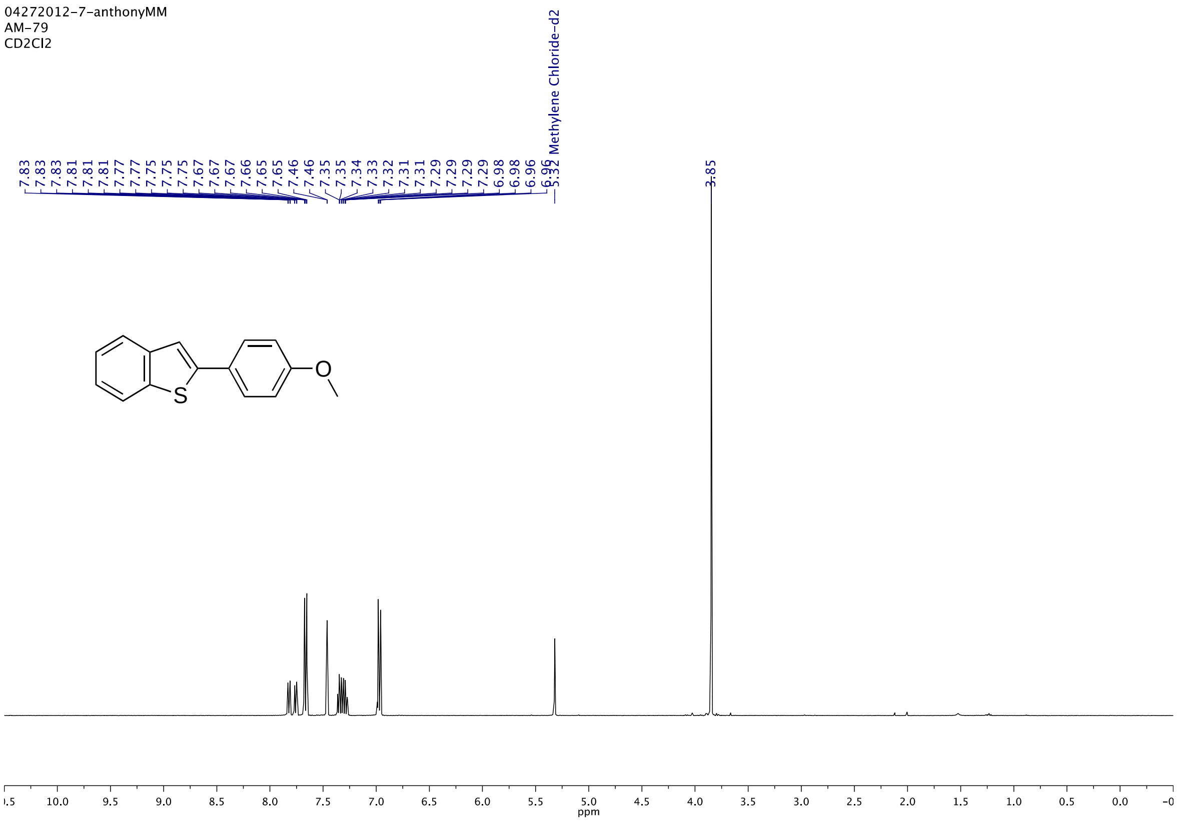
04272012-7-anthonyMM
AM-79
CD2Cl2
7.83 7.83 7.83 7.81 7.81 7.81 7.77 7.77 7.75 7.75 7.75 7.67 7.67 7.66 7.65 7.65 7.46 7.46 7.35 7.35 7.34 7.33 7.32 7.31 7.31 7.29 7.29 7.29 7.29 6.98 6.98 6.96 6.96
5.32 Methylene Chloride-d2
3.85
S
O
ppm

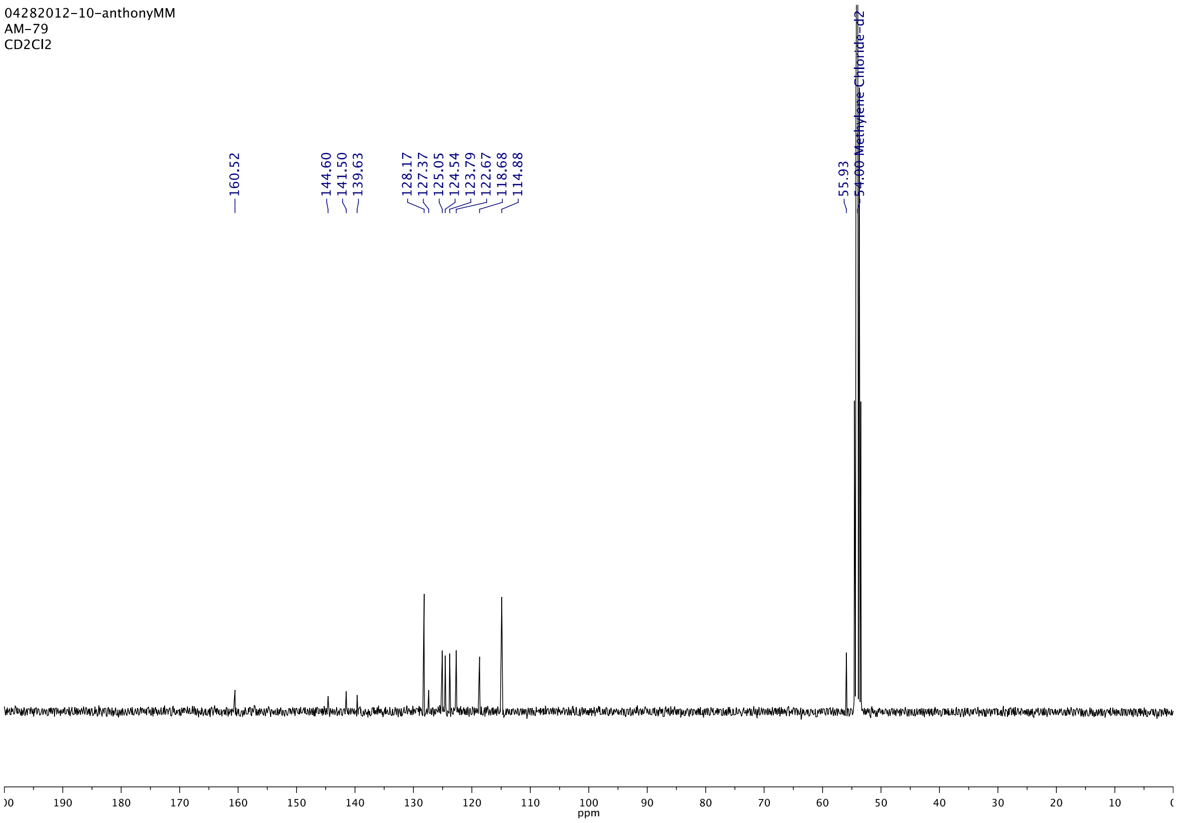
04282012-10-anthonyMM
AM-79
CD2Cl2
160.52
144.60 141.50 139.63
128.17 127.37 125.05 124.54 123.79 122.67 118.68 114.88
55.93
54.00 Methylene Chloride-d2
ppm

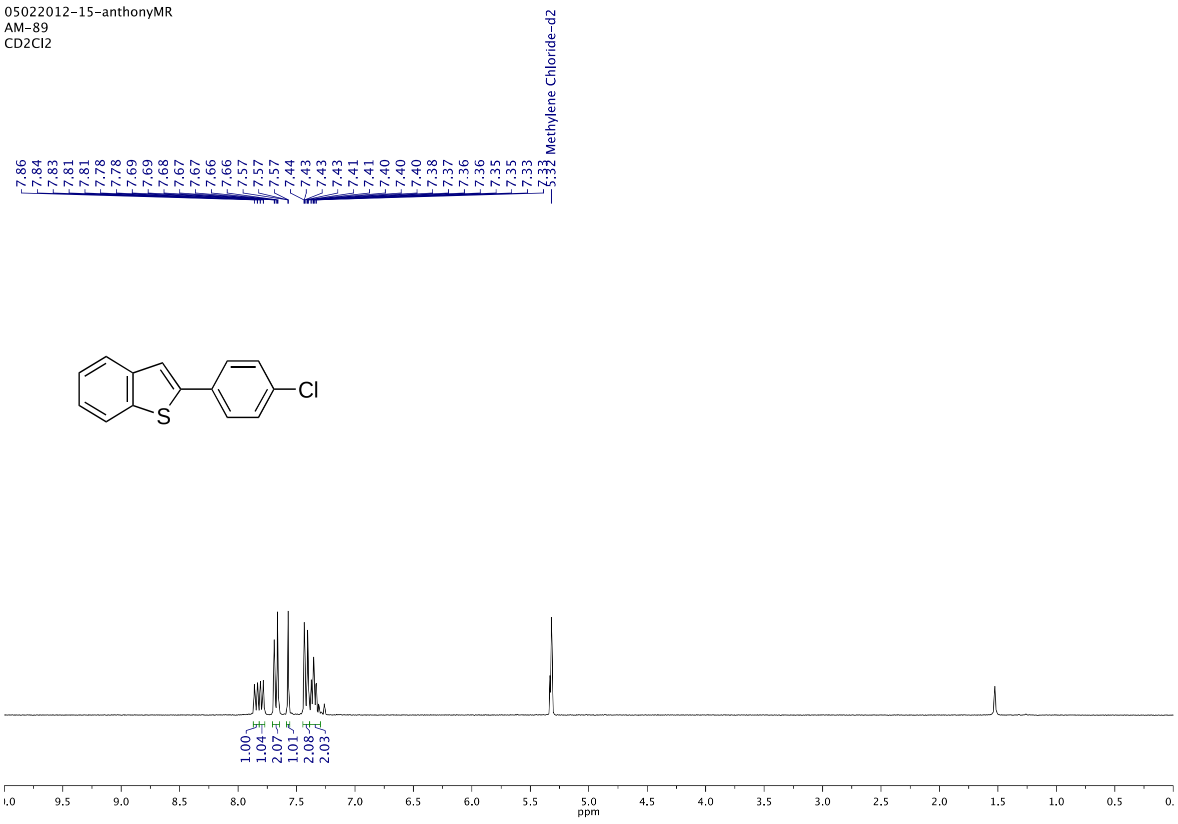
05022012-15-anthonyMR
AM-89
CD2Cl2
7.86 7.84 7.83 7.81 7.81 7.78 7.78 7.69 7.69 7.68 7.67 7.67 7.66 7.66 7.57 7.57 7.57 7.44 7.43 7.43 7.43 7.41 7.41 7.40 7.40 7.40 7.38 7.37 7.36 7.36 7.35 7.35 7.33 7.33
5.32 Methylene Chloride-d2
S
Cl
1.00 1.04 2.07 1.01 2.08 2.03
ppm

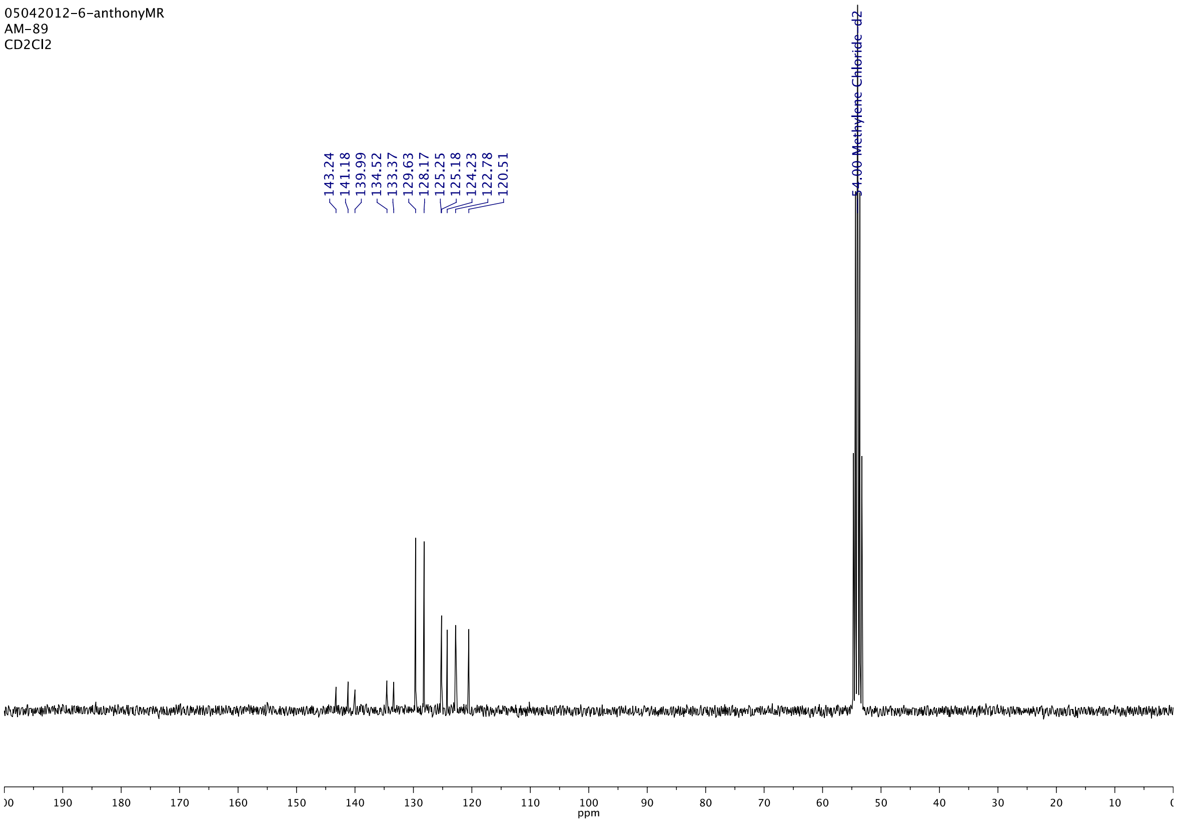
05042012-6-anthonyMR
AM-89
CD2Cl2
143.24 141.18 139.99 134.52 133.37 129.63 128.17 125.25 125.18 124.23 122.78 120.51
54.00 Methylene Chloride-d2
ppm

05052012-26-anthonyMR
AM-94
CD2Cl2

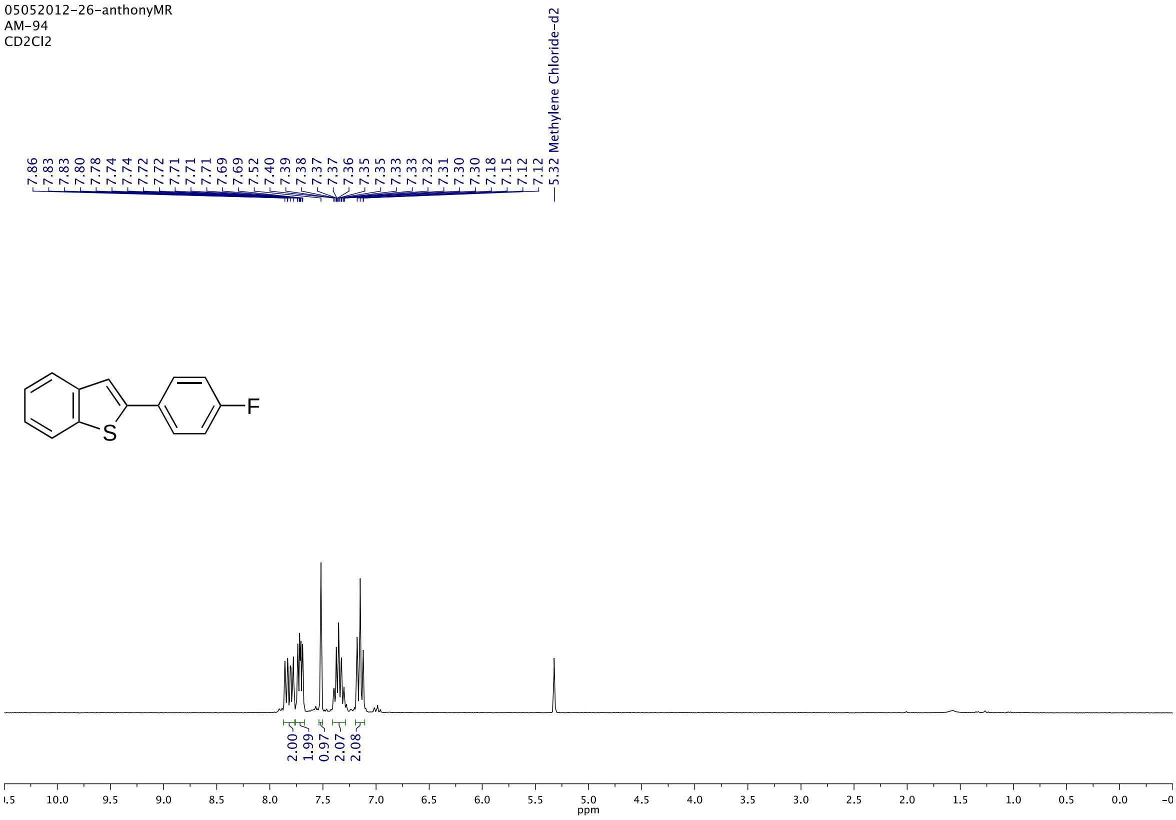

05052012-28-anthonyMR
AM-94
CD2Cl2

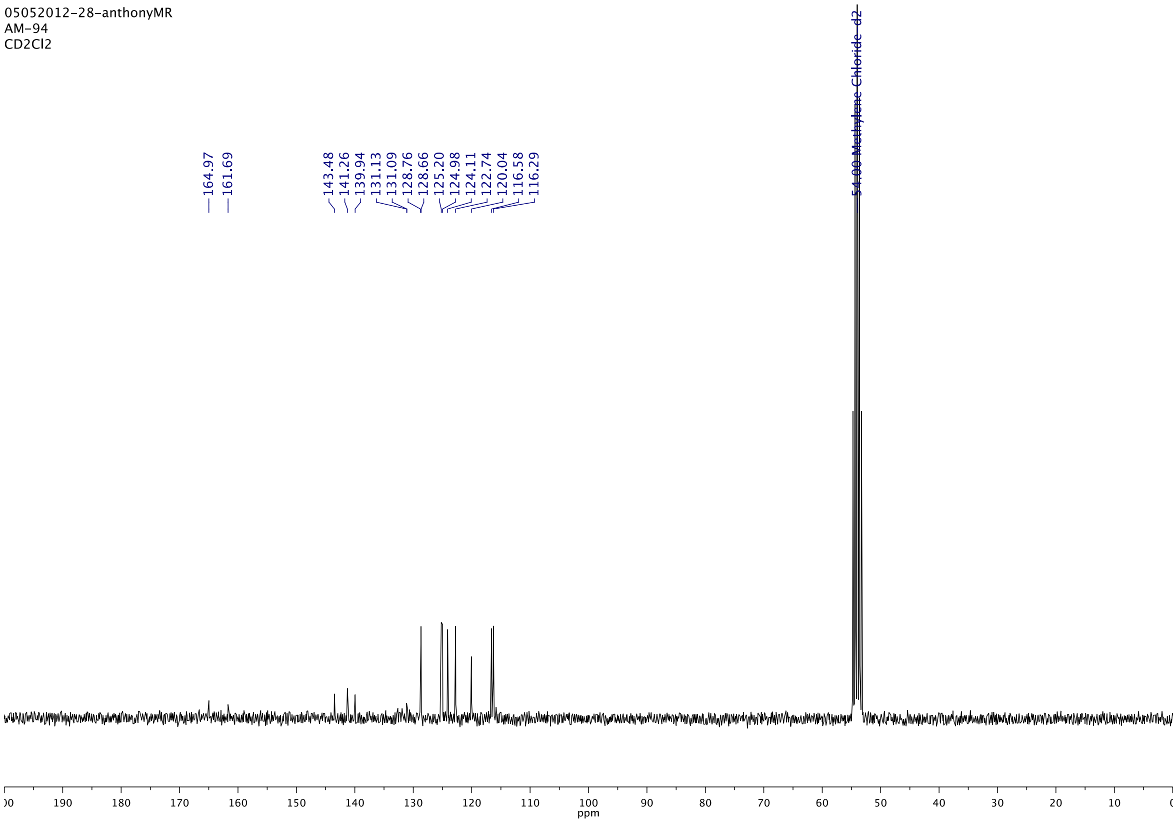

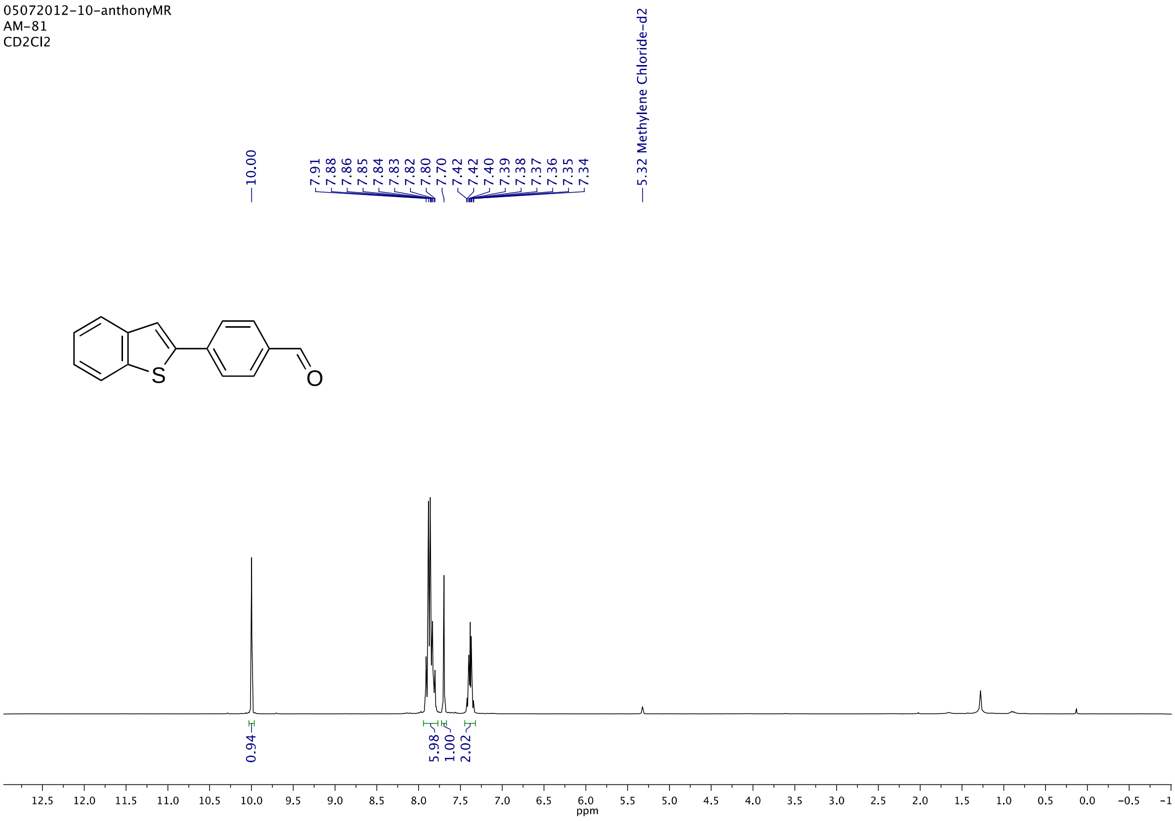
05072012-10-anthonyMR
AM-81
CD2Cl2
10.00
7.91 7.88 7.86 7.85 7.84 7.83 7.82 7.80 7.70 7.42 7.42 7.40 7.39 7.38 7.37 7.36 7.35 7.34
5.32 Methylene Chloride-d2
S
O
0.94
5.98
1.00
2.02
ppm

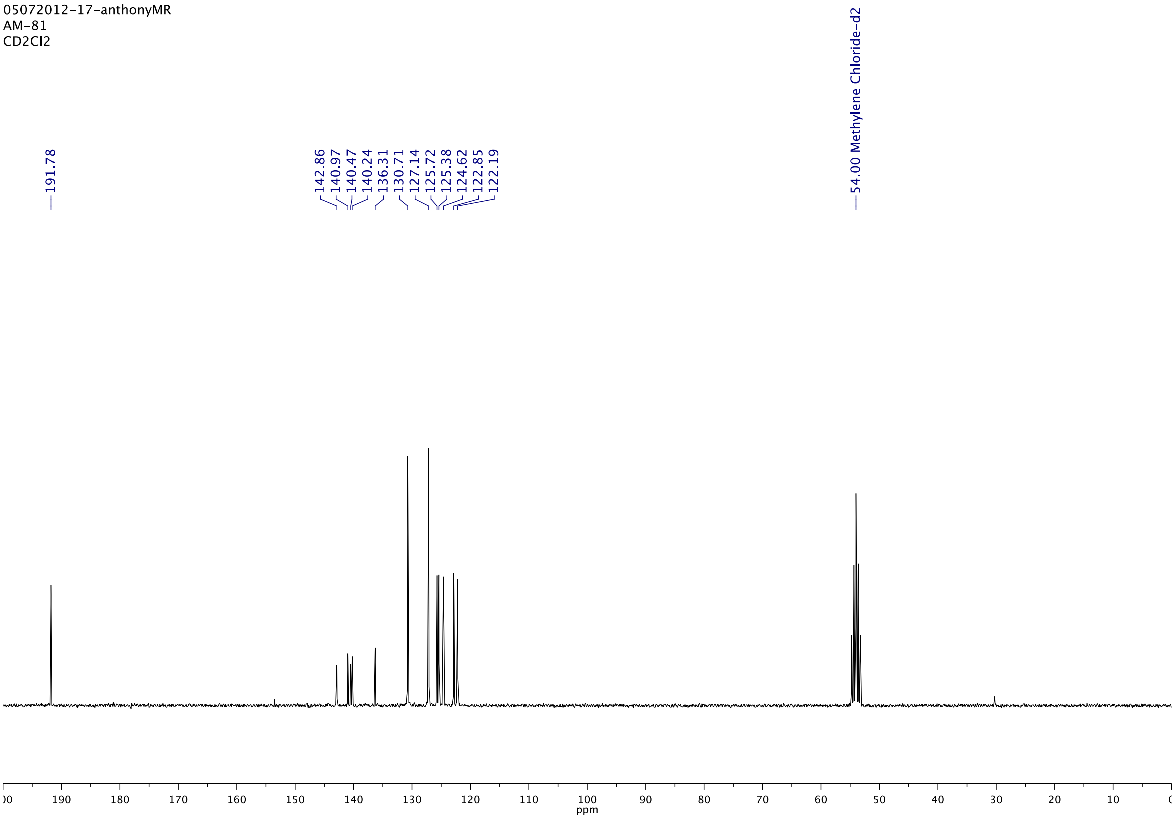
05072012-17-anthonyMR
AM-81
CD2Cl2
191.78
142.86 140.97 140.47 140.24 136.31 130.71 127.14 125.72 125.38 124.62 122.85 122.19
54.00 Methylene Chloride-d2
ppm

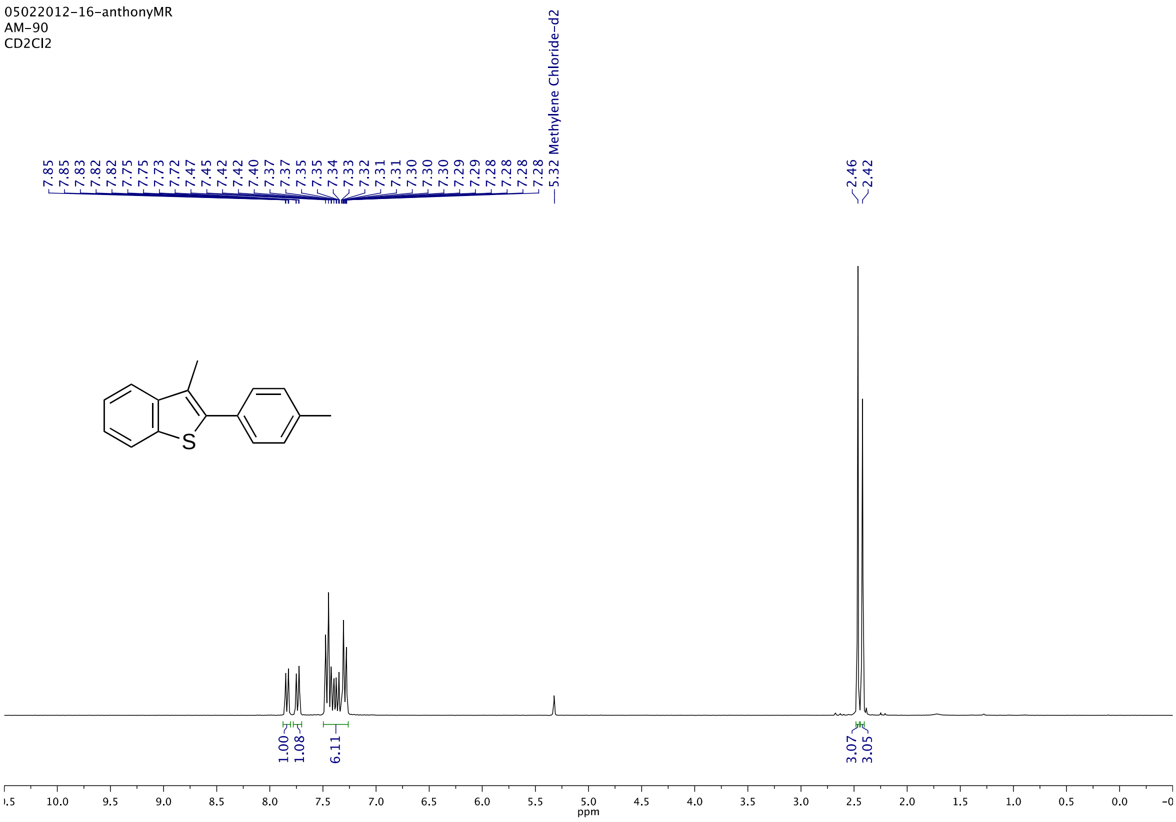
05022012-16-anthonyMR
AM-90
CD2Cl2
7.85 7.85 7.83 7.82 7.82 7.75 7.75 7.73 7.72 7.47 7.45 7.42 7.42 7.40 7.37 7.37 7.35 7.35 7.34 7.33 7.32 7.31 7.31 7.30 7.30 7.30 7.29 7.29 7.28 7.28 7.28 7.28
5.32 Methylene Chloride-d2
2.46
2.42
S
1.00
1.08
6.11
3.07
3.05
ppm

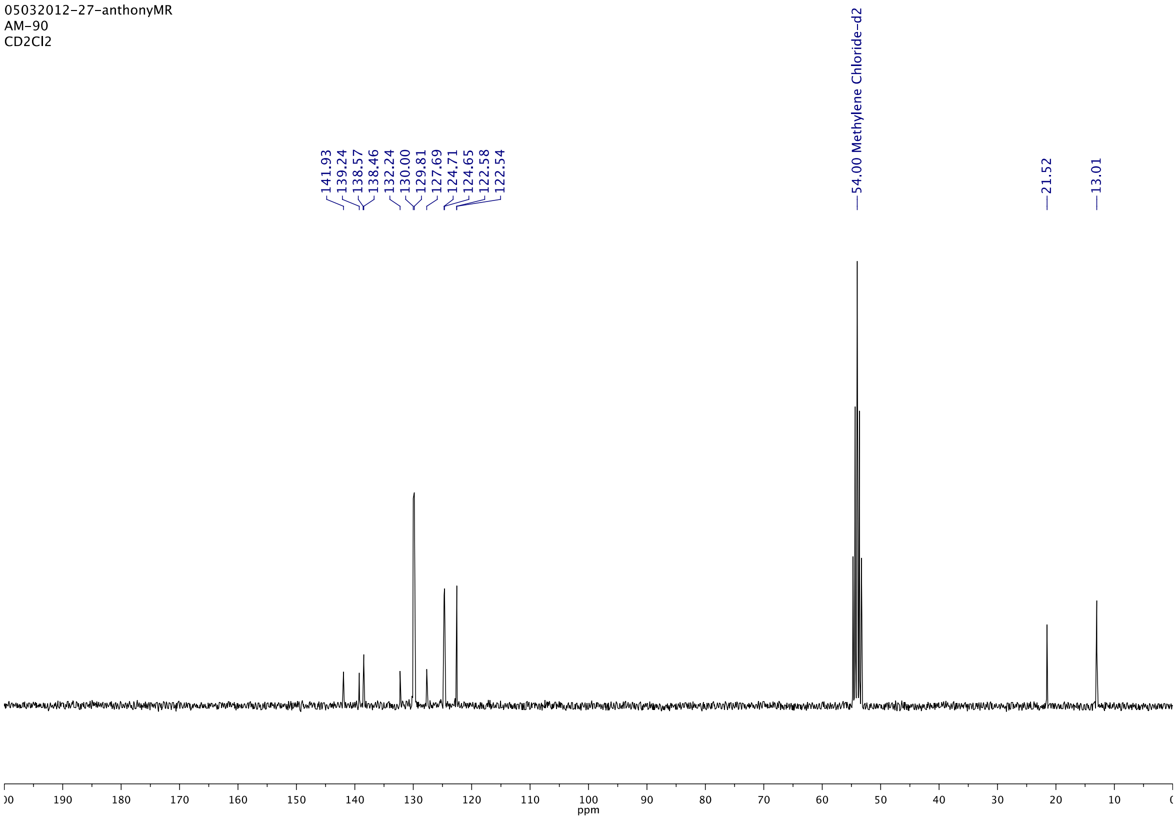
05032012-27-anthonyMR
AM-90
CD2Cl2
141.93 139.24 138.57 138.46 132.24 130.00 129.81 127.69 124.71 124.65 122.58 122.54
54.00 Methylene Chloride-d2
21.52
13.01
ppm

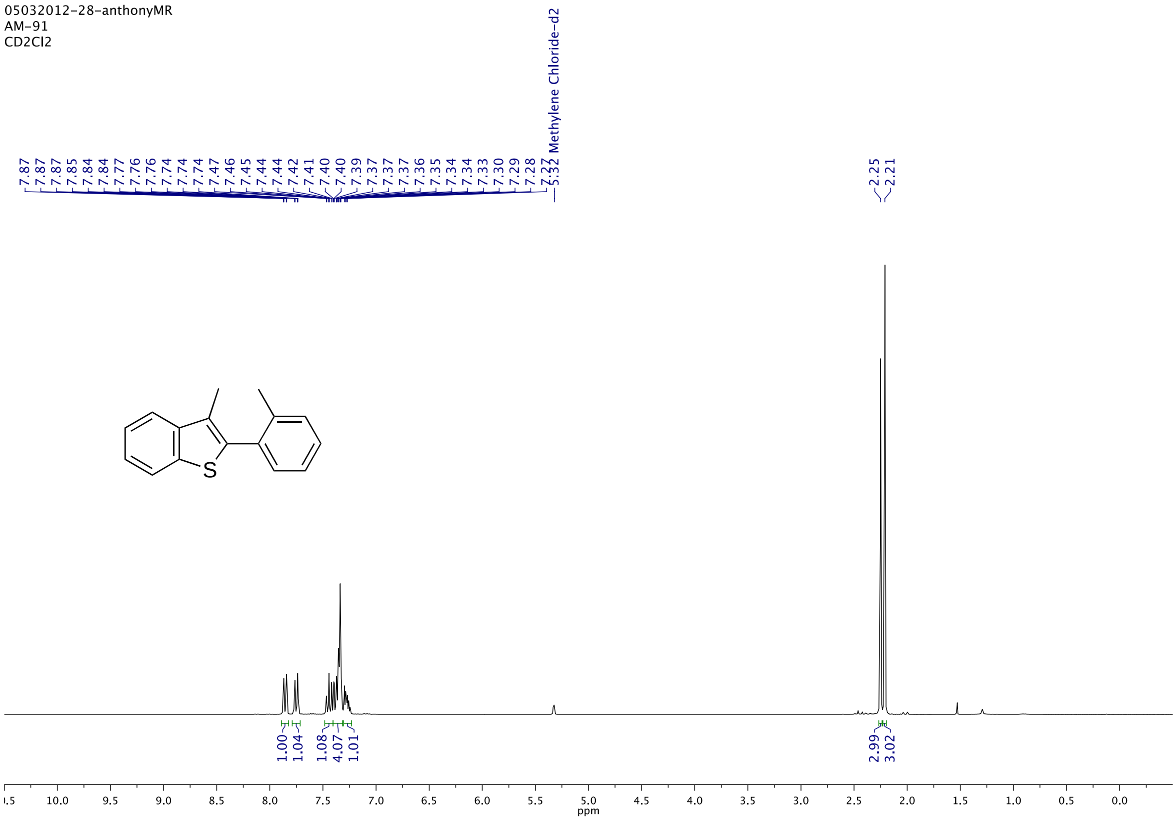
05032012-28-anthonyMR
AM-91
CD2Cl2
7.87 7.87 7.87 7.85 7.84 7.84 7.77 7.76 7.76 7.74 7.74 7.74 7.47 7.46 7.45 7.44 7.44 7.42 7.41 7.40 7.40 7.39 7.37 7.37 7.37 7.36 7.35 7.34 7.34 7.33 7.30 7.29 7.28 7.27
5.32 Methylene Chloride-d2
2.25
2.21
S
1.00
1.04
1.08
4.07
1.01
2.99
3.02
ppm

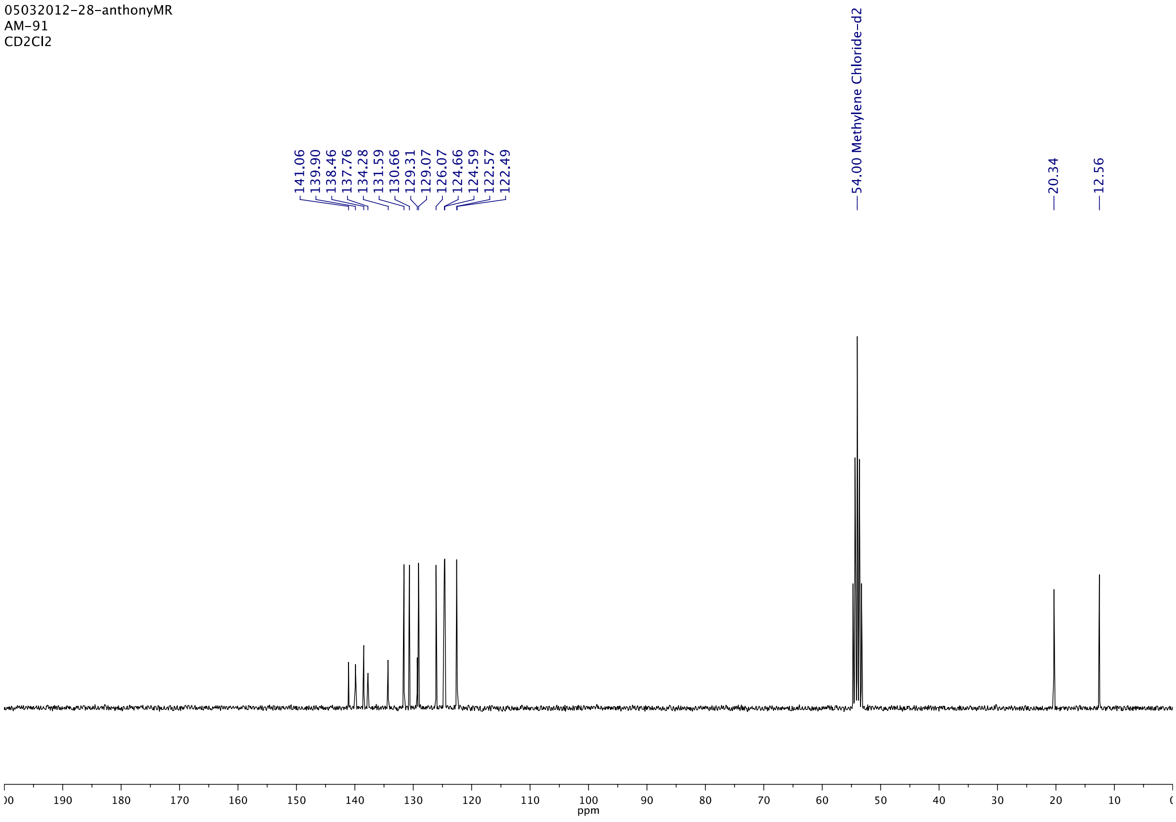
05032012-28-anthonyMR
AM-91
CD2Cl2
141.06 139.90 138.46 137.76 134.28 131.59 130.66 129.31 129.07 126.07 124.66 124.59 122.57 122.49
54.00 Methylene Chloride-d2
20.34
12.56
ppm

05042012-5-anthonyMR
AM-92
CD2Cl2

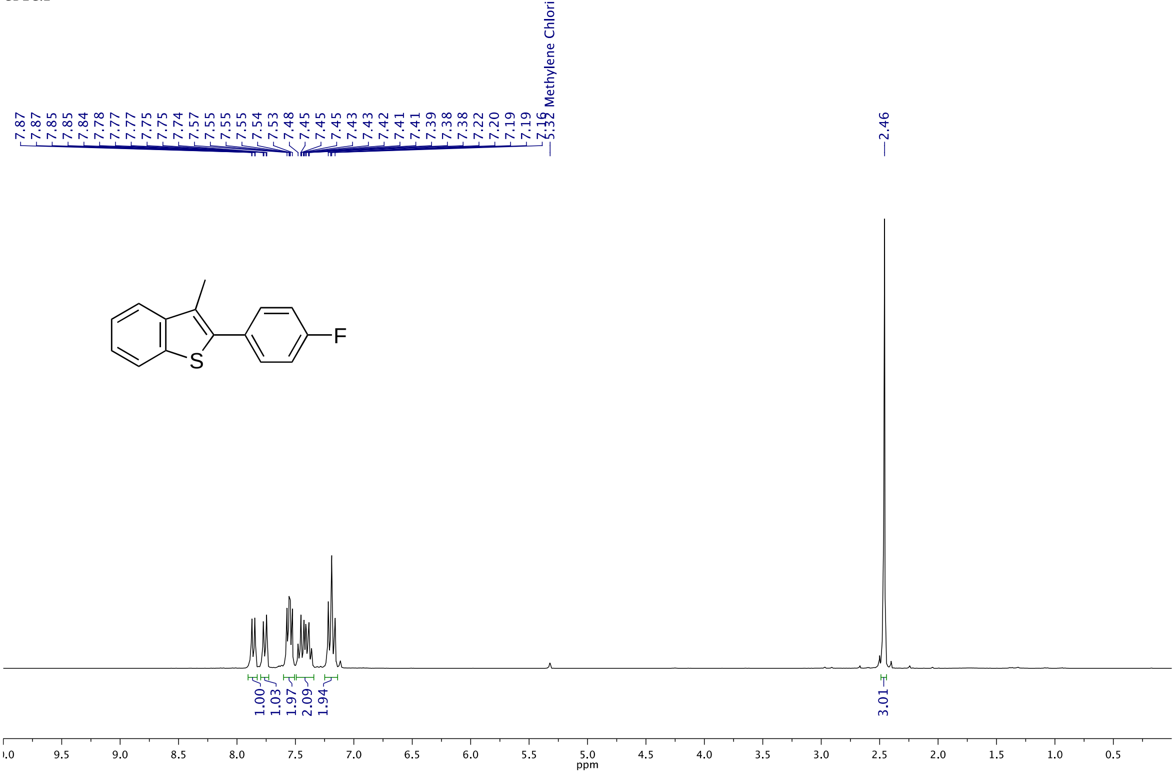

05052012-16-anthonyMM
AM-92
CD2Cl2

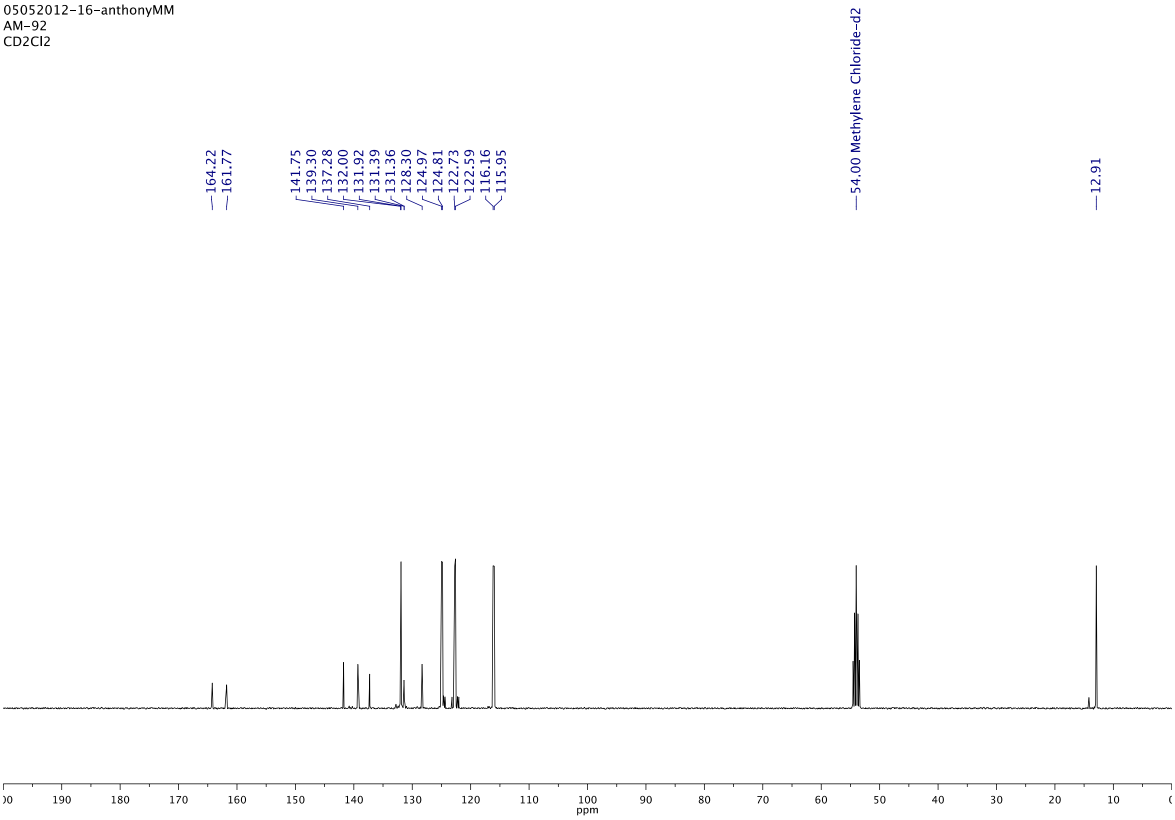

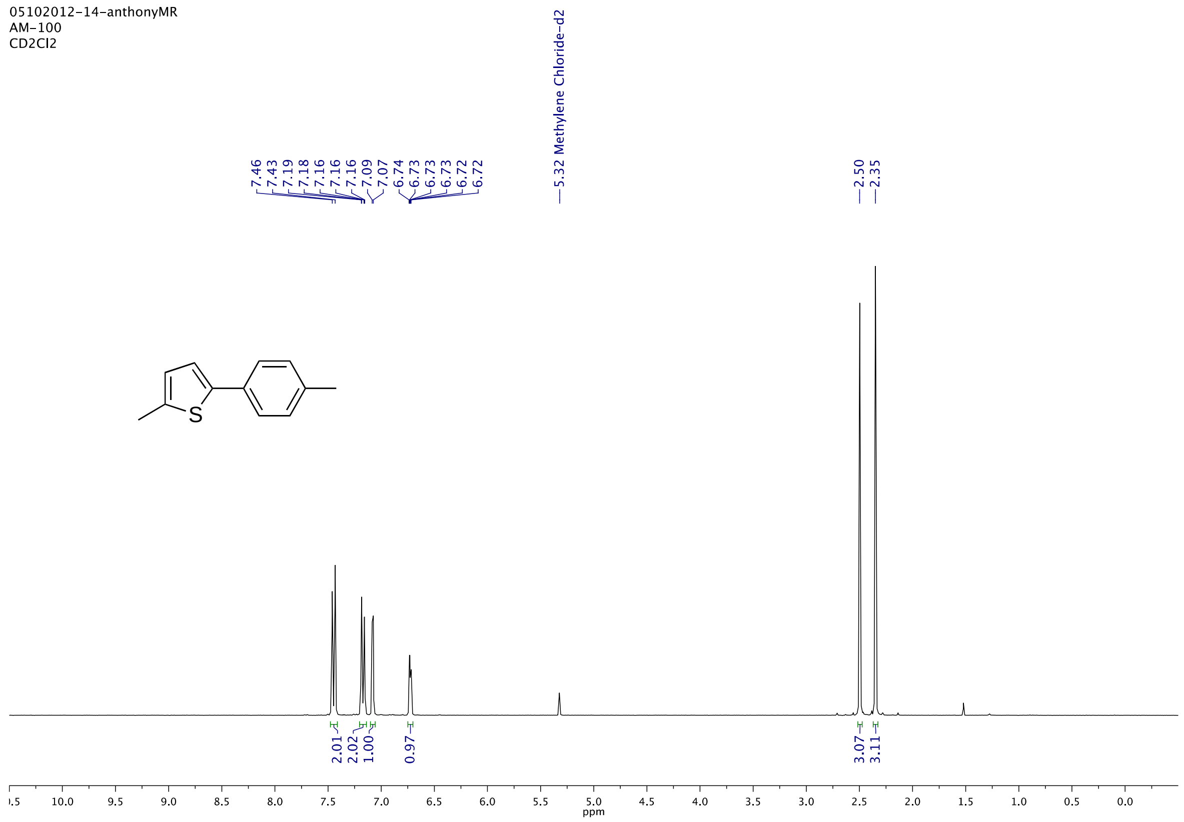
05102012-14-anthonyMR
AM-100
CD2Cl2
7.46
7.43
7.19
7.18
7.16
7.16
7.16
7.09
7.07
6.74
6.73
6.73
6.73
6.72
6.72
5.32 Methylene Chloride-d2
2.50
2.35
S
2.01
2.02
1.00
0.97
3.07
3.11
10.0
9.5
9.0
8.5
8.0
7.5
7.0
6.5
6.0
5.5
5.0
4.5
4.0
3.5
3.0
2.5
2.0
1.5
1.0
0.5
0.0
ppm

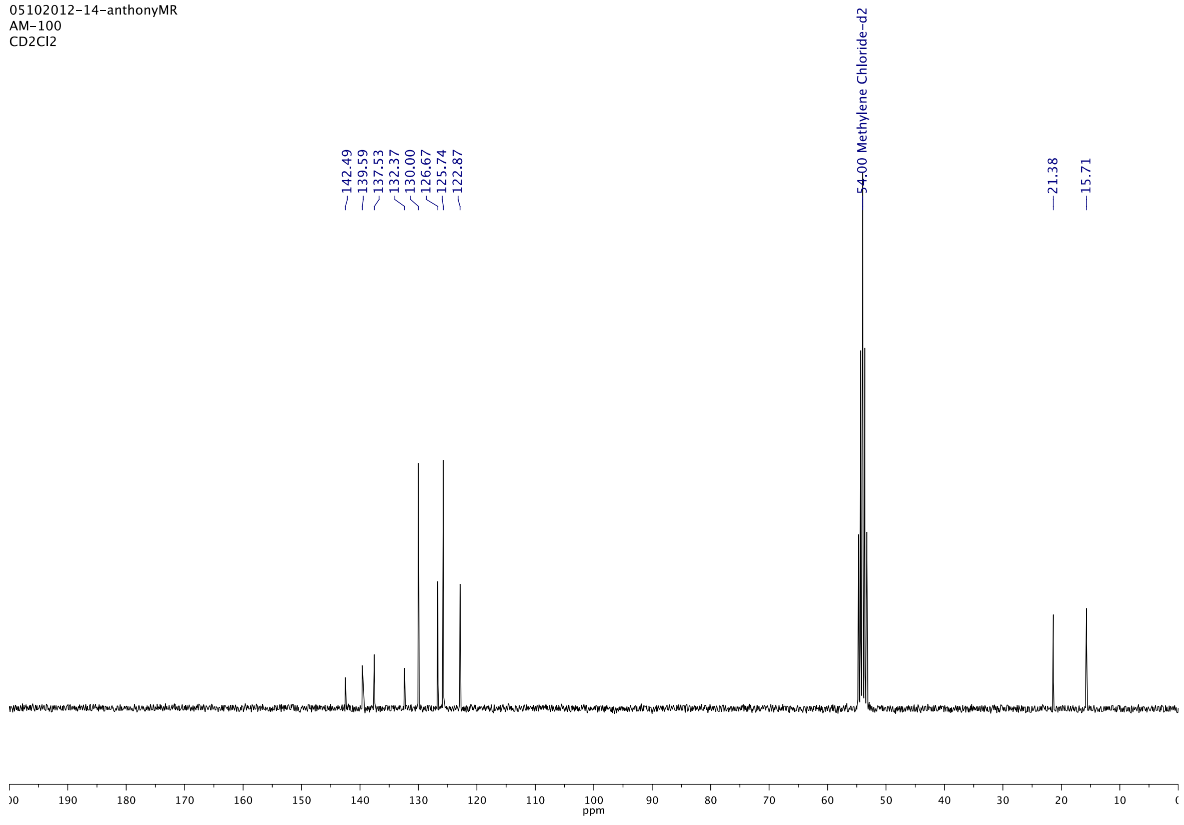
05102012-14-anthonyMR
AM-100
CD2Cl2
142.49
139.59
137.53
132.37
130.00
126.67
125.74
122.87
54.00 Methylene Chloride-d2
21.38
15.71
190
180
170
160
150
140
130
120
110
100
90
80
70
60
50
40
30
20
10
ppm

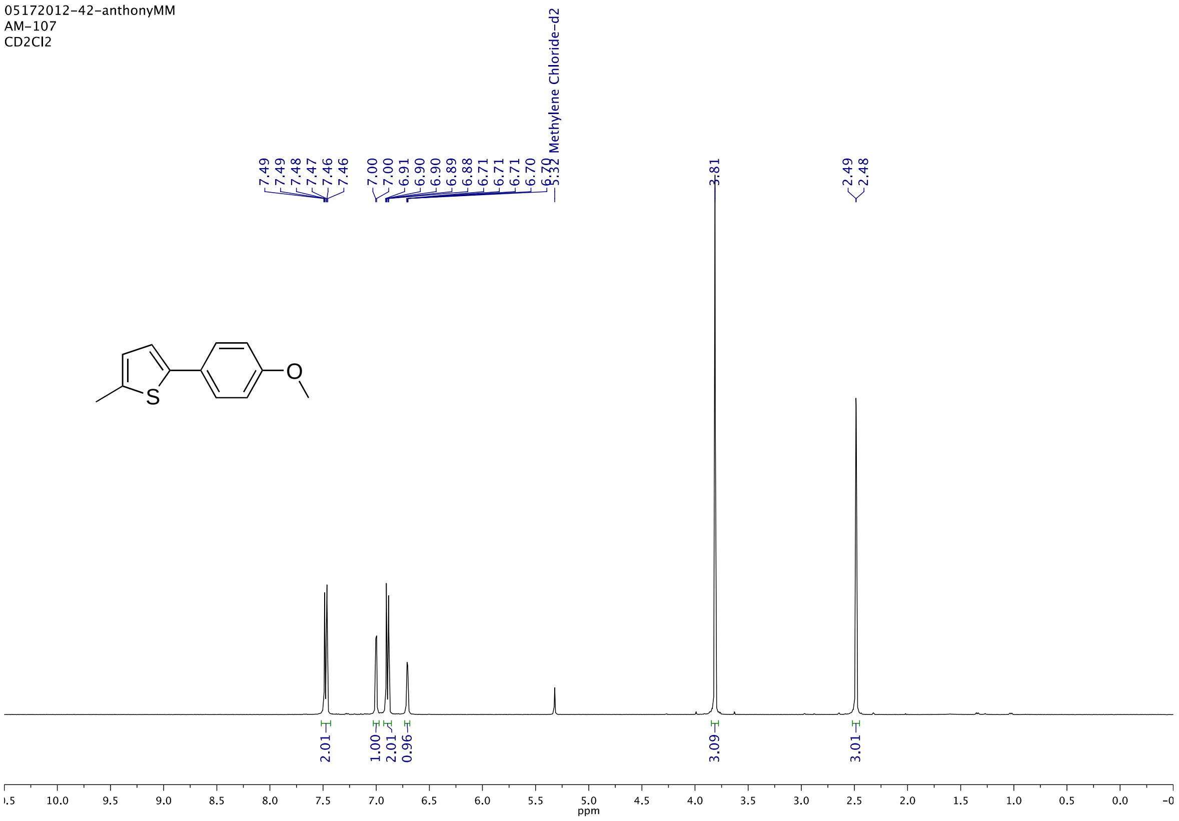
05172012-42-anthonyMM
AM-107
CD2Cl2
7.49
7.49
7.48
7.47
7.46
7.46
7.00
7.00
6.91
6.90
6.90
6.89
6.88
6.71
6.71
6.71
6.70
6.70
5.32 Methylene Chloride-d2
3.81
2.49
2.48
S
O
2.01
1.00
2.01
0.96
3.09
3.01
10.0 9.5 9.0 8.5 8.0 7.5 7.0 6.5 6.0 5.5 5.0 4.5 4.0 3.5 3.0 2.5 2.0 1.5 1.0 0.5 0.0
ppm

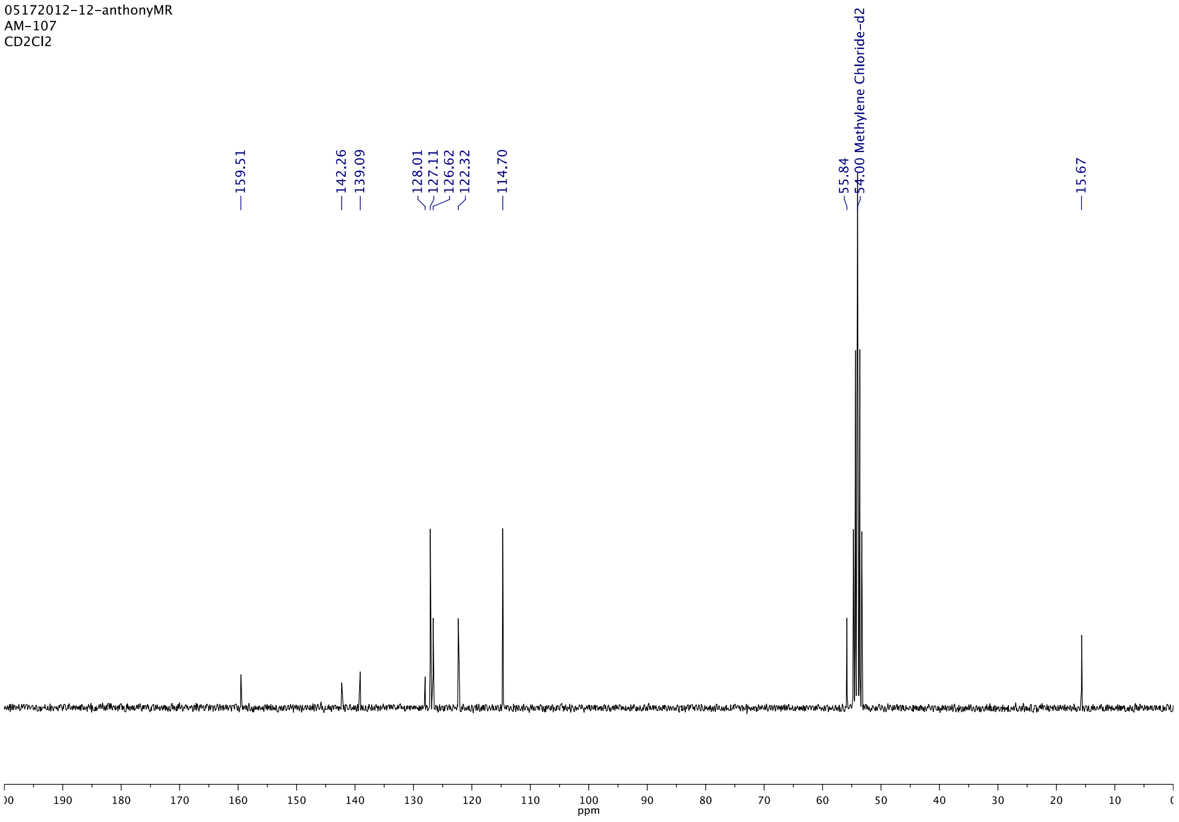
05172012-12-anthonyMR
AM-107
CD2Cl2
159.51
142.26
139.09
128.01
127.11
126.62
122.32
114.70
55.84
54.00 Methylene Chloride-d2
15.67
190 180 170 160 150 140 130 120 110 100 90 80 70 60 50 40 30 20 10
ppm

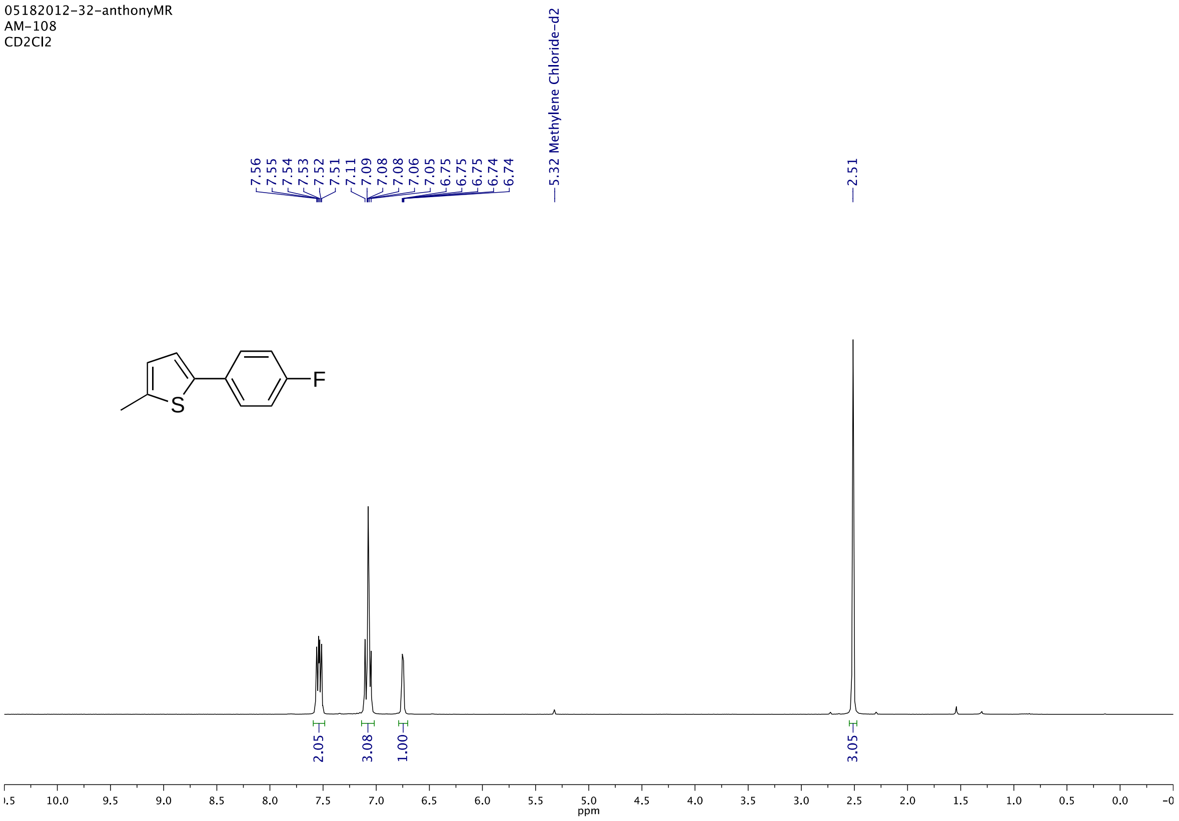
05182012-32-anthonyMR
AM-108
CD2Cl2
7.56
7.55
7.54
7.53
7.52
7.51
7.11
7.09
7.08
7.08
7.06
7.05
6.75
6.75
6.74
6.74
5.32 Methylene Chloride-d2
2.51
F
S
2.05
3.08
1.00
3.05
ppm

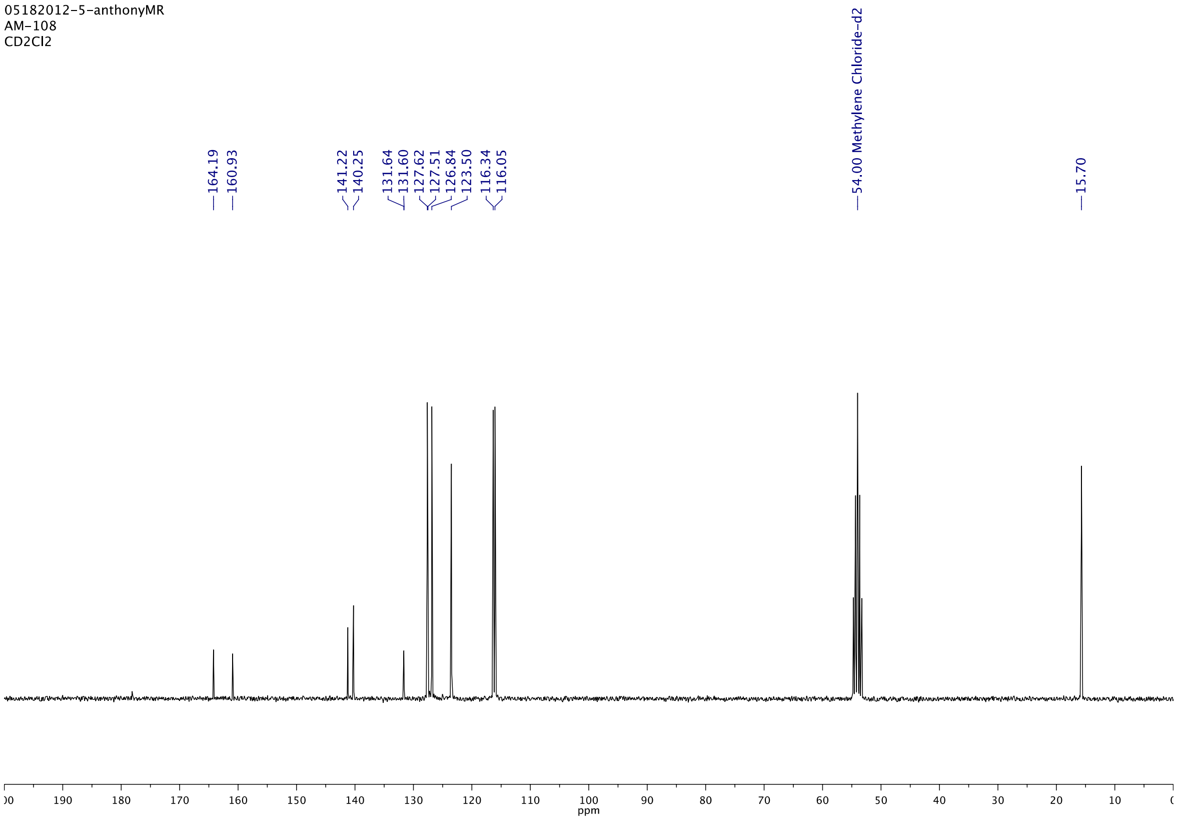
05182012-5-anthonyMR
AM-108
CD2Cl2
164.19
160.93
141.22
140.25
131.64
131.60
127.62
127.51
126.84
123.50
116.34
116.05
54.00 Methylene Chloride-d2
15.70
ppm

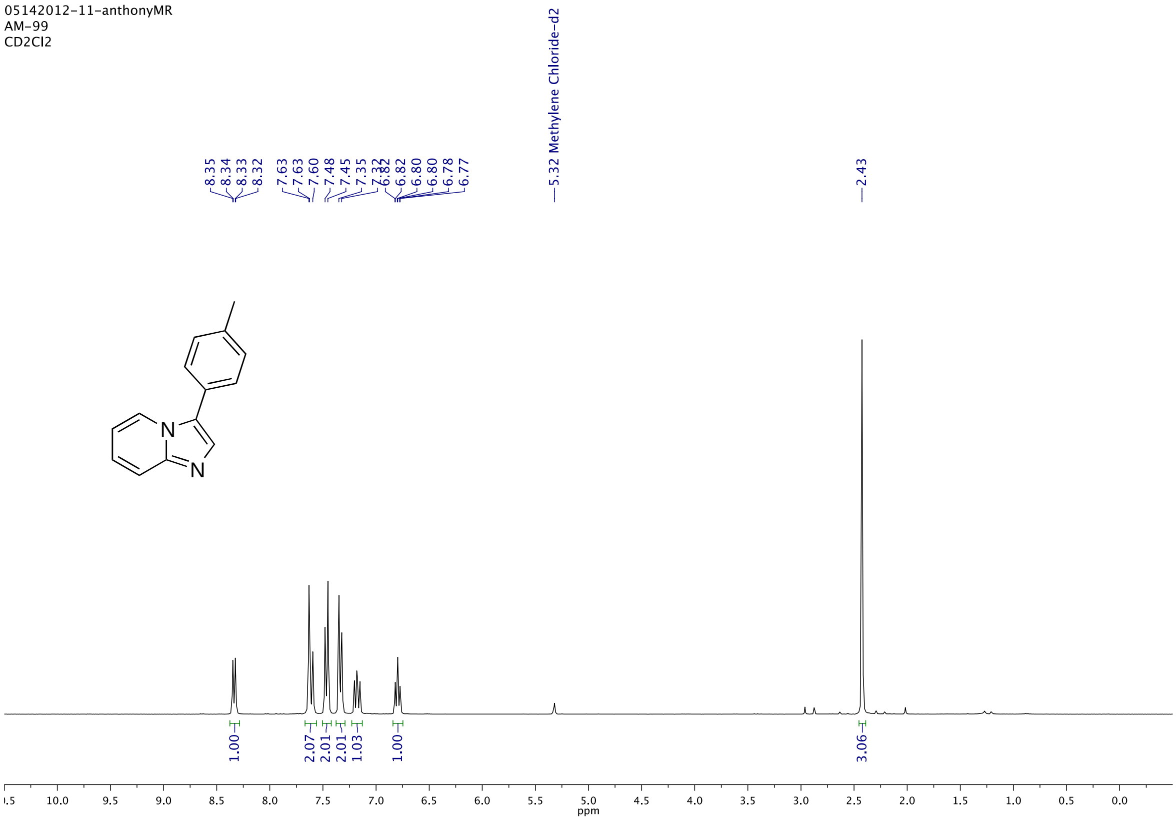
05142012-11-anthonyMR
AM-99
CD2Cl2
8.35
8.34
8.33
8.32
7.63
7.63
7.60
7.48
7.45
7.35
7.32
6.82
6.82
6.80
6.80
6.78
6.77
5.32 Methylene Chloride-d2
2.43
N
N
1.00
2.07
2.01
2.01
1.03
1.00
3.06
10.0
9.5
9.0
8.5
8.0
7.5
7.0
6.5
6.0
5.5
5.0
4.5
4.0
3.5
3.0
2.5
2.0
1.5
1.0
0.5
0.0
ppm

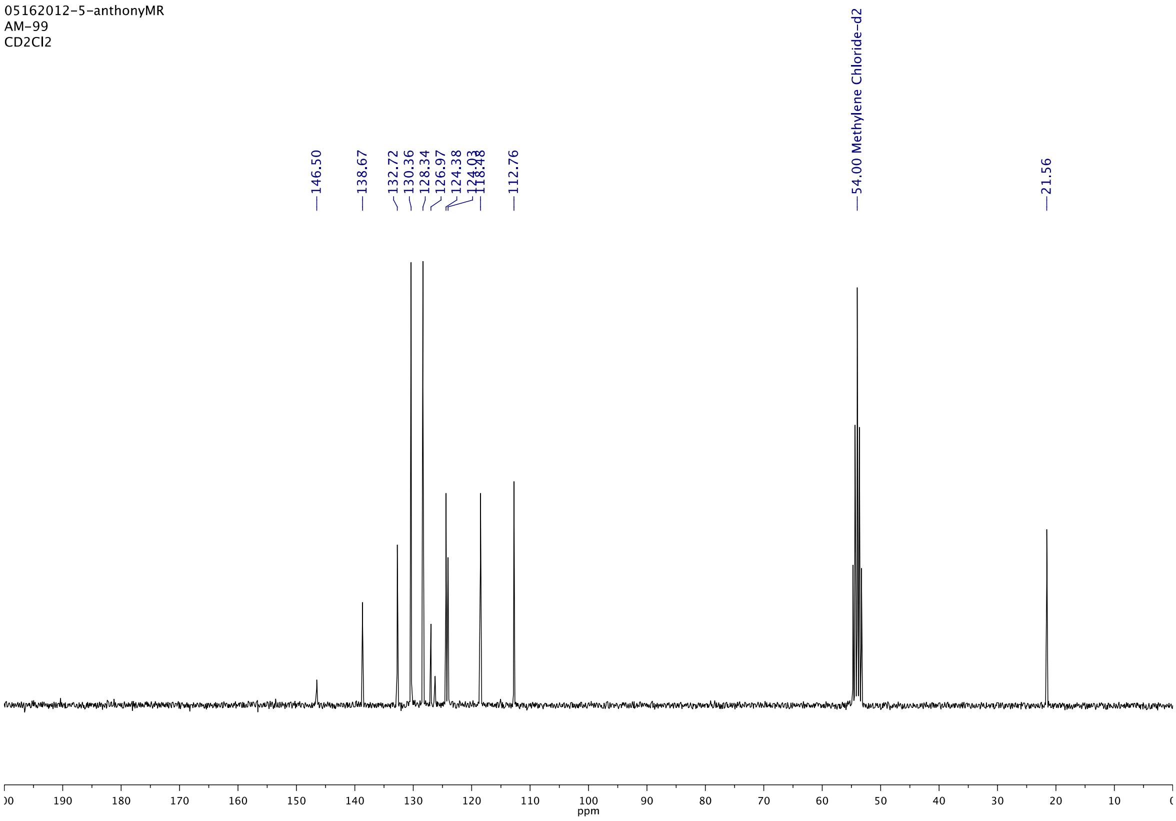
05162012-5-anthonyMR
AM-99
CD2Cl2
146.50
138.67
132.72
130.36
128.34
126.97
124.38
124.03
118.48
112.76
54.00 Methylene Chloride-d2
21.56
190
180
170
160
150
140
130
120
110
100
90
80
70
60
50
40
30
20
10
ppm

05162012-10-anthonyMI
AM-104
CD2Cl2

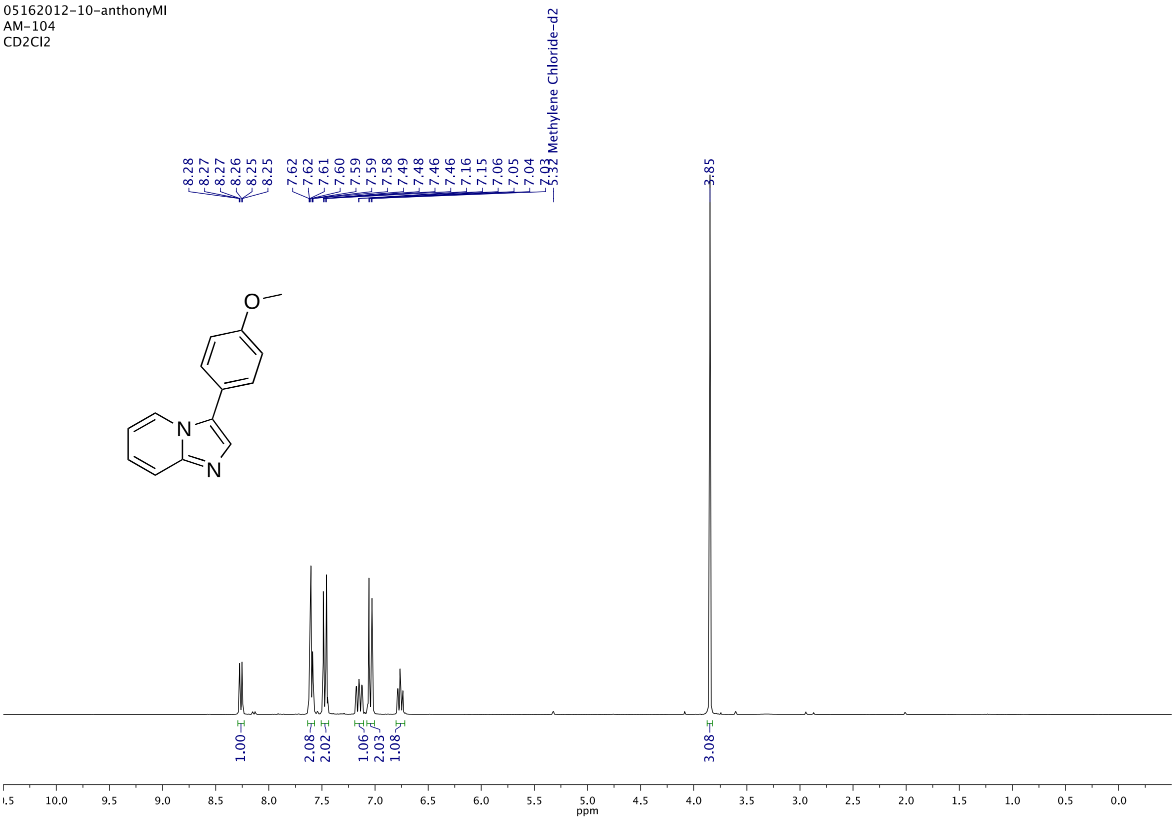

05162012-6-anthonyMR
AM-104
CD2Cl2

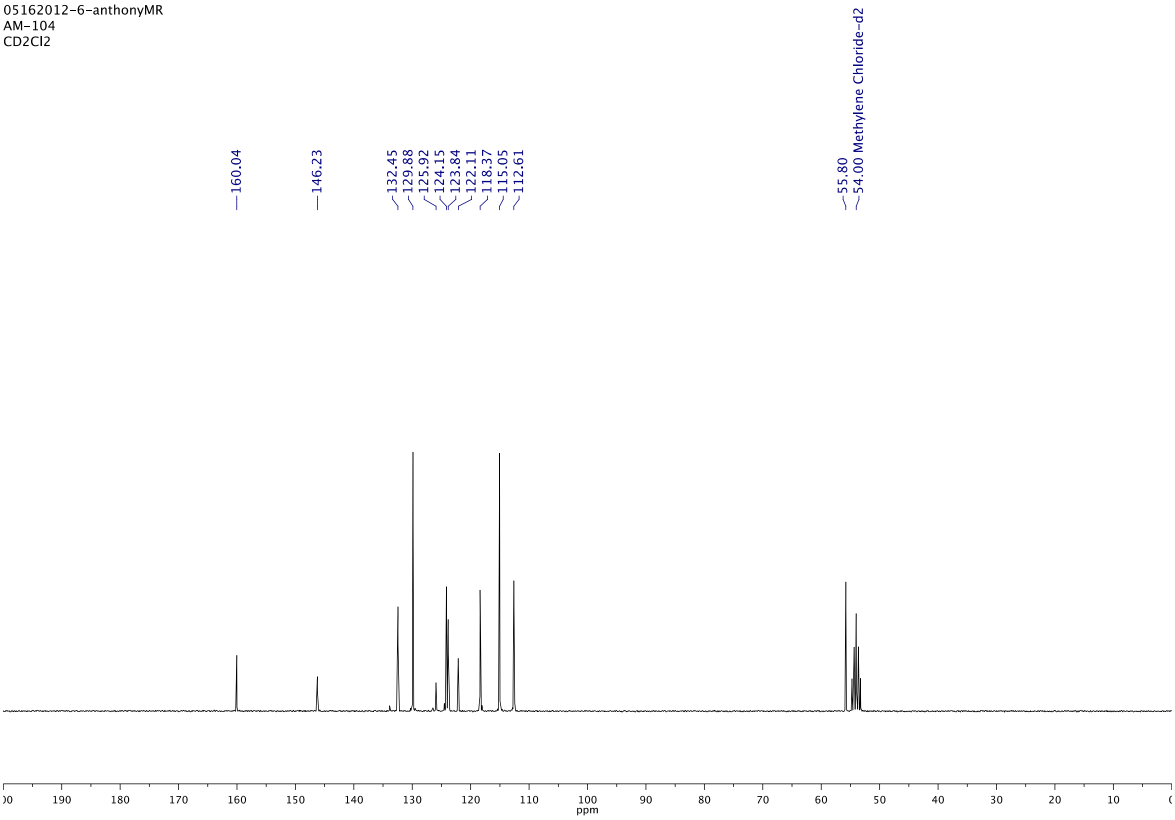

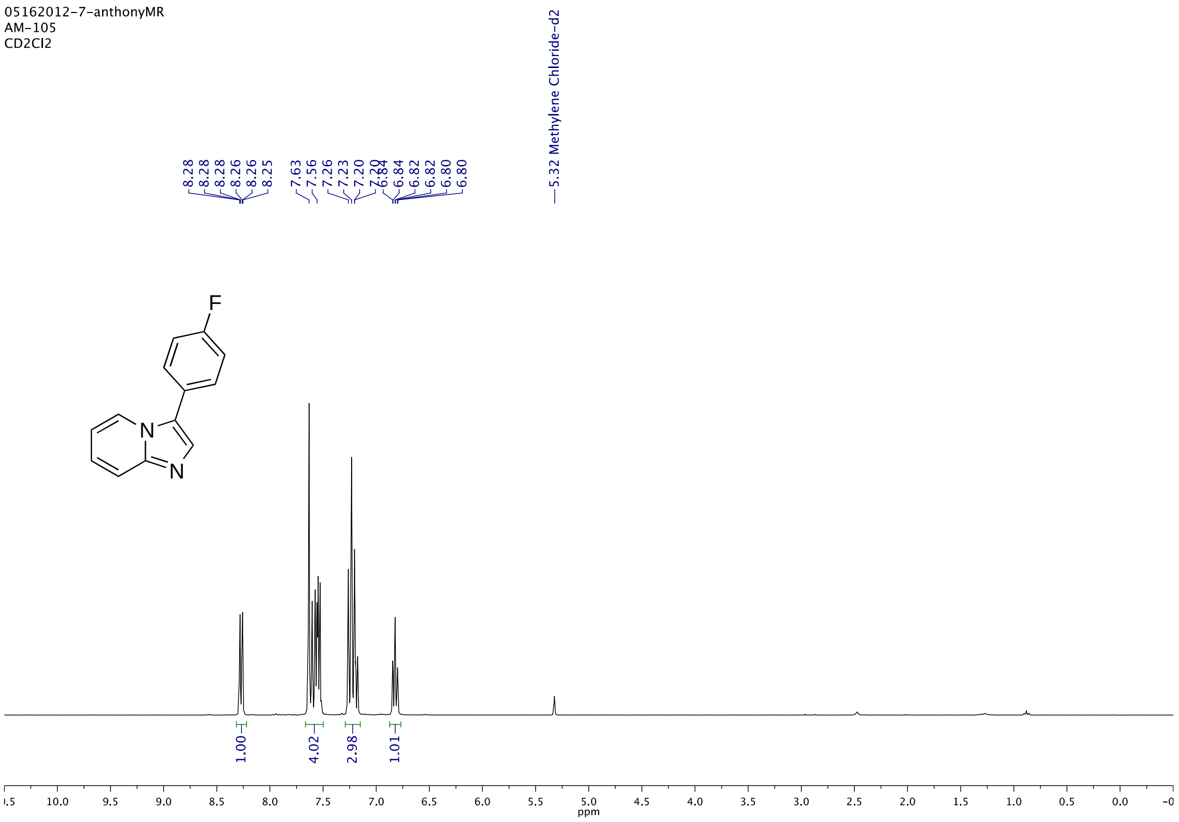
05162012-7-anthonyMR
AM-105
CD2Cl2
8.28
8.28
8.28
8.26
8.26
8.25
7.63
7.56
7.26
7.23
7.20
7.20
6.84
6.84
6.82
6.82
6.80
6.80
5.32 Methylene Chloride-d2
F
N
N
1.00
4.02
2.98
1.01
ppm

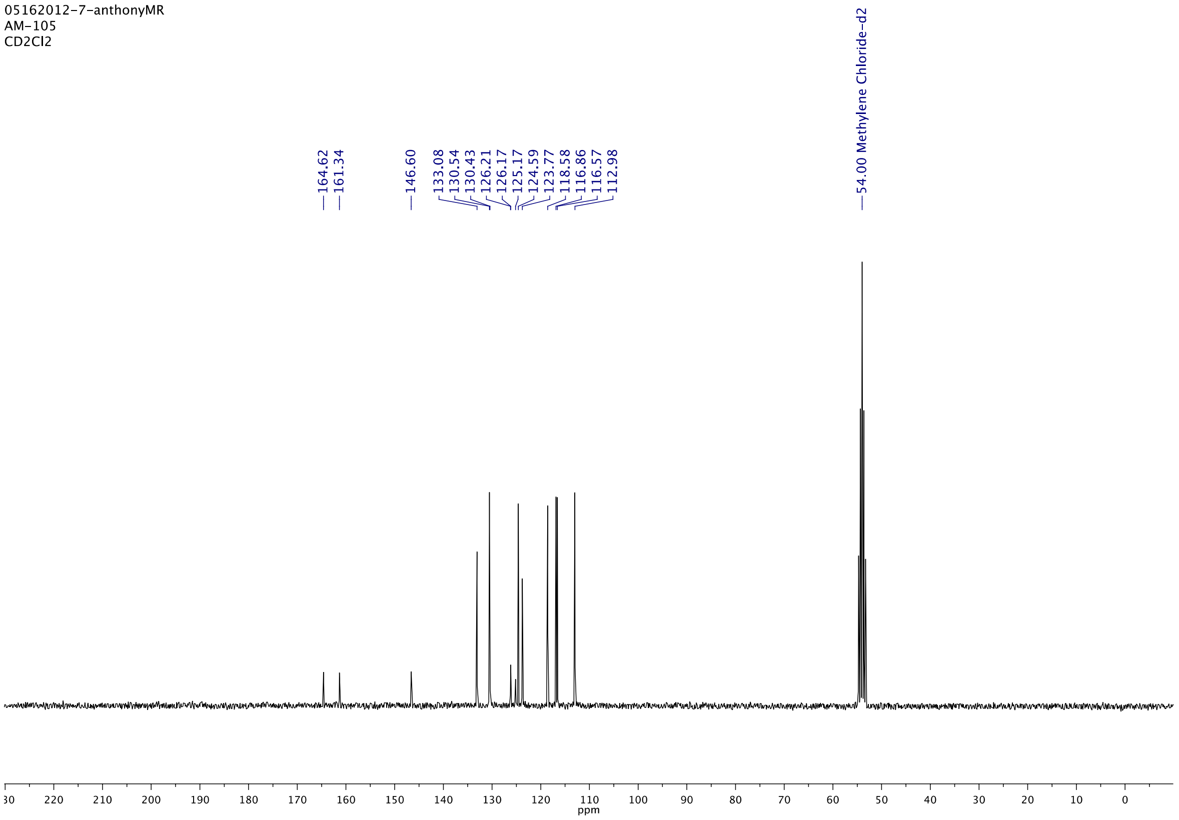
05162012-7-anthonyMR
AM-105
CD2Cl2
164.62
161.34
146.60
133.08
130.54
130.43
126.21
126.17
125.17
124.59
123.77
118.58
116.86
116.57
112.98
54.00 Methylene Chloride-d2
ppm
