# Supplementary material for: Extending the utility of [Pd(NHC)(cinnamyl)Cl] precatalysts: Direct arylation of heterocycles
Source: Beilstein J Org Chem. 2012 Sep 27;8:1637–43. doi: 10.3762/bjoc.8.187 (PMC3510996; doi:10.3762/bjoc.8.187)

Supporting Information

for

Extending the utility of [Pd(NHC)(cinnamyl)Cl] pre-catalysts: Direct arylation of heterocycles

Anthony R. Martin, Anthony Chartoire, Alexandra M. Z. Slawin and Steven P. Nolan*

Address: EastCHEM School of Chemistry, University of St Andrews, North Haugh, St Andrews, KY16 9ST, UK

Email: Steven P. Nolan* - snolan@st-andrews.ac.uk

* Corresponding author

CIF-Check for compound 4

checkCIF/PLATON report

Structure factors have been supplied for datablock(s) 4

No syntax errors found. CIF dictionary Interpreting this report

Datablock: 4

Bond precision: C-C = 0.0110 A Wavelength=0.71075

Cell:	a=13.8017(7)	b=17.0279(9)	c=18.9988(14)
	alpha=89.971(7)	beta=80.984(7)	gamma=70.150(8)
Temperature:	93 K		

	Calculated	Reported
Volume	4141.1(5)	4141.1(5)
Space group	P -1	P -1
Hall group	-P 1	-P 1
Moiety formula	2(C86 H81 Cl N2 Pd), C5 H11, C H2 Cl2, C H3	C89.50 H89 Cl2 N2 Pd
Sum formula	C179 H178 Cl4 N4 Pd2	C89.50 H89 Cl2 N2 Pd
Mr	2739.86	1370.01
Dx,g cm-3	1.099	1.099
Z	1	2
Mu (mm-1)	0.330	0.330
F000	1440.0	1440.0
F000’	1439.04	
h,k,lmax	16,20,22	16,20,22
Nref	15175	15028
Tmin,Tmax	0.980,0.980	0.789,1.000
Tmin’	0.980	

Correction method= MULTI-SCAN

Data completeness= 0.990 Theta(max)= 25.350

R(reflections)= 0.0843(12236) wR2(reflections)= 0.2577(15028)

S = 1.043 Npar= 880

The following ALERTS were generated. Each ALERT has the format
test-name_ALERT_alert-type_alert-level.
Click on the hyperlinks for more details of the test.

Alert level A

PLAT241_ALERT_2_A Check High Ueq as Compared to Neighbors for C70

Author Response: Correct atom identies, established by other chemical means.

```
PLAT241_ALERT_2_A Check High      Ueq as Compared to Neighbors for        C71
```

Author Response: Correct atom identies, established by other chemical means.

```
PLAT241_ALERT_2_A Check High      Ueq as Compared to Neighbors for        C83
```

Author Response: Correct atom identies, established by other chemical means.

```
PLAT241_ALERT_2_A Check High      Ueq as Compared to Neighbors for        C86
```

Author Response: Correct atom identies, established by other chemical means.

Alert level B

```
PLAT220_ALERT_2_B Large Non-Solvent    C     Ueq(max)/Ueq(min) ...        7.2 Ratio
PLAT230_ALERT_2_B Hirshfeld Test Diff for    C82    --  C83     ..       15.1 su
PLAT230_ALERT_2_B Hirshfeld Test Diff for    C83    --  C84     ..       18.2 su
PLAT232_ALERT_2_B Hirshfeld Test Diff (M-X)  Pd1    --  C83     ..       20.8 su
PLAT234_ALERT_4_B Large Hirshfeld Difference C86    --  C87     ..       0.26 Ang.
PLAT242_ALERT_2_B Check Low       Ueq as Compared to Neighbors for        C84
PLAT601_ALERT_2_B Structure Contains Solvent Accessible VOIDS of .        154 A**3
PLAT910_ALERT_3_B Missing # of FCF Reflections Below Th(Min) .....         28
PLAT934_ALERT_3_B Number of (Iobs-Icalc)/SigmaW .gt. 10 Outliers .          5
```

Alert level C

```
RFACR01_ALERT_3_C  The value of the weighted R factor is > 0.25
            Weighted R factor given   0.258
PLAT042_ALERT_1_C Calc. and Reported MoietyFormula Strings  Differ          ?
PLAT045_ALERT_1_C Calculated and Reported Z Differ by ...........       0.50 Ratio
PLAT084_ALERT_2_C High wR2 Value .................................      0.26
PLAT094_ALERT_2_C Ratio of Maximum / Minimum Residual Density ....      2.44
PLAT213_ALERT_2_C Atom C26              has ADP max/min Ratio .....      3.1 prola
PLAT213_ALERT_2_C Atom C70              has ADP max/min Ratio .....      3.3 prola
PLAT213_ALERT_2_C Atom C71              has ADP max/min Ratio .....      3.4 prola
PLAT213_ALERT_2_C Atom C86              has ADP max/min Ratio .....      4.0 prola
PLAT213_ALERT_2_C Atom C87              has ADP max/min Ratio .....      3.9 prola
PLAT222_ALERT_3_C Large Non-Solvent    H    Uiso(max)/Uiso(min) ..      6.7 Ratio
PLAT234_ALERT_4_C Large Hirshfeld Difference C23    --  C24     ..       0.16 Ang.
PLAT234_ALERT_4_C Large Hirshfeld Difference C76    --  C77     ..       0.18 Ang.
PLAT241_ALERT_2_C Check High      Ueq as Compared to Neighbors for        C22
```

Author Response: Correct atom identies, established by other chemical means.

```
PLAT241_ALERT_2_C Check High      Ueq as Compared to Neighbors for        C77
```

Author Response: Correct atom identies, established by other chemical means.

```
PLAT241_ALERT_2_C Check High      Ueq as Compared to Neighbors for        C82
```

Author Response: Correct atom identies, established by other chemical means.

```
PLAT241_ALERT_2_C Check High      Ueq as Compared to Neighbors for        C87
```

Author Response: Correct atom identies, established by other chemical means.

```
PLAT242_ALERT_2_C Check Low       Ueq as Compared to Neighbors for        Pd1
PLAT242_ALERT_2_C Check Low       Ueq as Compared to Neighbors for        C66
PLAT242_ALERT_2_C Check Low       Ueq as Compared to Neighbors for        C69
PLAT242_ALERT_2_C Check Low       Ueq as Compared to Neighbors for        C85
PLAT242_ALERT_2_C Check Low       Ueq as Compared to Neighbors for        C89
PLAT334_ALERT_2_C Small Average Benzene  C-C Dist. C66    -C71         1.37 Ang.
PLAT342_ALERT_3_C Low Bond Precision on  C-C Bonds ...............   0.0110 Ang
PLAT411_ALERT_2_C Short Inter H...H Contact  H70    ..  H71     ..     2.13 Ang.
PLAT911_ALERT_3_C Missing # FCF Refl Between THmin & STh/L=  0.600       89
PLAT912_ALERT_4_C Missing # of FCF Reflections Above STh/L=  0.600       33
PLAT913_ALERT_3_C Missing # of Very Strong Reflections in FCF ....        1
PLAT918_ALERT_3_C Reflection(s) # with I(obs) much smaller I(calc)       10
PLAT939_ALERT_3_C Large Value of Not (SHELXL) Weight Optimized S .    12.31
PLAT971_ALERT_2_C Large Calcd. Non-Metal Positive Residual Density     2.33 eA-3
PLAT971_ALERT_2_C Large Calcd. Non-Metal Positive Residual Density     1.52 eA-3
```

Alert level G

```
PLAT005_ALERT_5_G No _iucr_refine_instructions_details in CIF ....        ?
PLAT072_ALERT_2_G SHELXL First  Parameter in WGHT Unusually Large.     0.15
PLAT083_ALERT_2_G SHELXL Second Parameter in WGHT Unusually Large.     8.70
PLAT244_ALERT_4_G Low   'Solvent' Ueq as Compared to Neighbors of       C93
PLAT302_ALERT_4_G Note: Anion/Solvent Disorder ..................       100 Perc.
PLAT303_ALERT_2_G Full Occupancy H-Atom  H81A   with # Connections     2.00
PLAT303_ALERT_2_G Full Occupancy H-Atom  H83    with # Connections     2.00
PLAT432_ALERT_2_G Short Inter X...Y Contact  C91    ..  C92     ..     1.77 Ang.
PLAT432_ALERT_2_G Short Inter X...Y Contact  C91    ..  C93     ..     2.86 Ang.
PLAT773_ALERT_2_G Check long C-C Bond in CIF: C91    --  C92     .     1.77 Ang.
PLAT790_ALERT_4_G Centre of Gravity not Within Unit Cell: Resd.  #        4
              C H3
PLAT860_ALERT_3_G Note: Number of Least-Squares Restraints .......       14
```

```
   4 ALERT level A = Most likely a serious problem - resolve or explain
   9 ALERT level B = A potentially serious problem, consider carefully
  32 ALERT level C = Check. Ensure it is not caused by an omission or oversight
  12 ALERT level G = General information/check it is not something unexpected

   2 ALERT type 1 CIF construction/syntax error, inconsistent or missing data
  37 ALERT type 2 Indicator that the structure model may be wrong or deficient
  10 ALERT type 3 Indicator that the structure quality may be low
   7 ALERT type 4 Improvement, methodology, query or suggestion
   1 ALERT type 5 Informative message, check
```

It is advisable to attempt to resolve as many as possible of the alerts in all categories. Often the minor alerts point to easily fixed oversights, errors and omissions in your CIF or refinement strategy, so attention to these fine details can be worthwhile. In order to resolve some of the more serious problems it may be necessary to carry out additional measurements or structure refinements. However, the purpose of your study may justify the reported deviations and the more serious of these should normally be commented upon in the discussion or experimental section of a paper or in the "special_details" fields of the CIF. checkCIF was carefully designed to identify outliers and unusual parameters, but every test has its limitations and alerts that are not important in a particular case may appear. Conversely, the absence of alerts does not guarantee there are no aspects of the results needing attention. It is up to the individual to critically assess their own results and, if necessary, seek expert advice.

Publication of your CIF in IUCr journals

A basic structural check has been run on your CIF. These basic checks will be run on all CIFs submitted for publication in IUCr journals (*Acta Crystallographica*, *Journal of Applied Crystallography*, *Journal of Synchrotron Radiation*); however, if you intend to submit to *Acta Crystallographica Section C* or *E*, you should make sure that full publication checks are run on the final version of your CIF prior to submission.

Publication of your CIF in other journals

Please refer to the *Notes for Authors* of the relevant journal for any special instructions relating to CIF submission.

PLATON version of 19/04/2012; check.def file version of 14/04/2012

Datablock 4 - ellipsoid plot

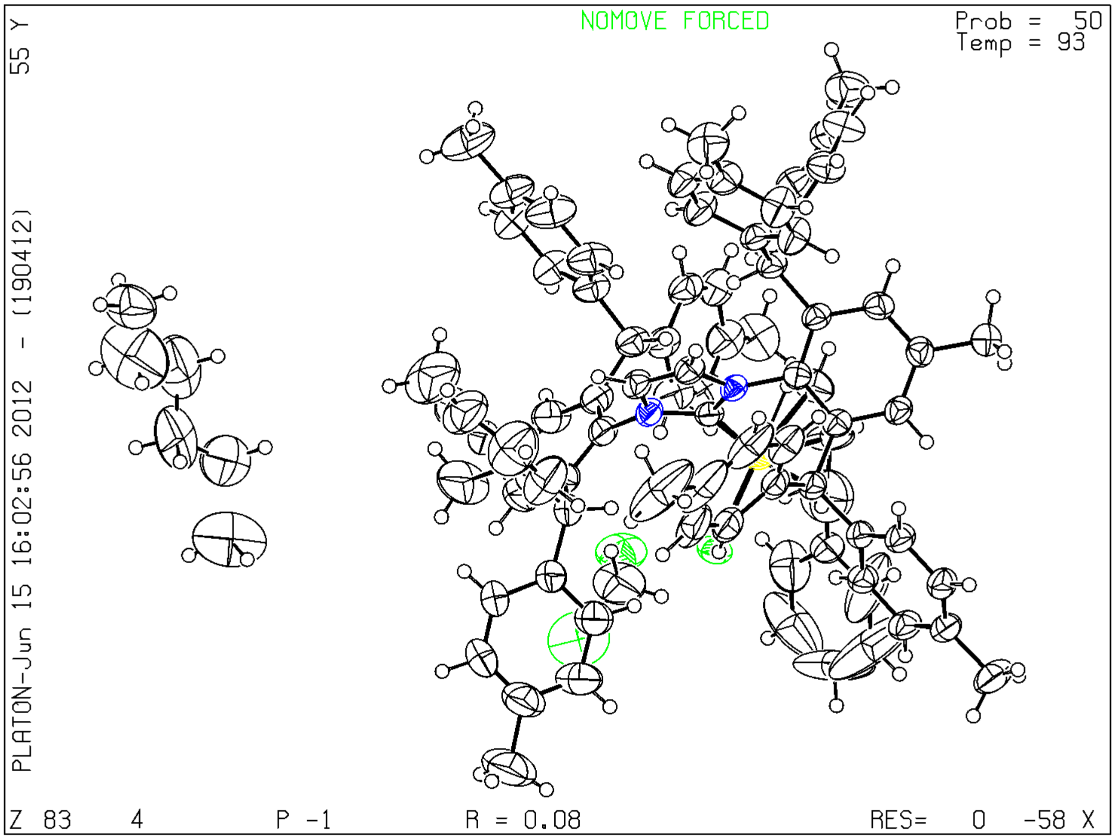

Supplement: File 2 — CIF-Check for compound 4. [file Beilstein_J_Org_Chem-08-1637-s002.pdf]
